# USP25 maintains KRAS expression and inhibiting the deubiquitinase suppresses KRAS signaling in human cancer

**DOI:** 10.1016/j.jbc.2025.110337

**Published:** 2025-06-03

**Authors:** Huailu Ma, Huiyuan Guan, Xiao Sun, Lingzhi Wu, Mengjiao Cai, Xinghua Zhen, Xiang Shen, Suxia Han, Guangxue Liu, Jin Peng, Pumin Zhang

**Affiliations:** 1 Zhejiang Provincial Key Laboratory of Pancreatic Disease, The First Affiliated Hospital of Zhejiang University, Hangzhou, Zhejiang, China; 2 Institute of Translational Medicine, Zhejiang University School of Medicine, Hangzhou, Zhejiang, China; 3 Cancer Center, Zhejiang University, Hangzhou, Zhejiang, China; 4 Department of Oncology, The First Affiliated Hospital, Xi'an Jiaotong University Medical College, Xi'an, China; 5 Chaser Therapeutics, Hangzhou, Zhejiang Province, China

**Keywords:** KRAS, USP25, ubiquitination, proteasomal degradation

## Abstract

*KRAS* is a prominent oncogene mutated in a large number of human malignancies, particularly in pancreatic, colorectal, and lung tumors. We demonstrate here that KRAS, including its various activating mutants, is subjected to ubiquitin-mediated proteasomal degradation in cancer cells. Through an siRNA-based screening of deubiquitinases, we identified USP25 as a deubiquitinase for KRAS. Depleting *USP25* expression increases ubiquitination and proteasomal degradation of KRAS, leading to the suppression of its oncogenic activity. We further show that USP25 inhibitors we have discovered are capable of destabilizing KRAS in cancer cells and are efficacious in blocking tumor xenograft growth in mice. These findings provide evidence supporting the notion that targeting the deubiquitinase USP25 can effectively, albeit indirectly, suppress KRAS and potentially aid in the treatment of tumors driven by KRAS-activating mutations.

The ubiquitin-proteasome system is critical in maintaining cellular protein homeostasis. Proteins destined for proteasomal degradation are usually labeled by polyubiquitin chains linked *via* the lysine 48 or the lysine 11 residues in ubiquitin (1, 2). This labeling process involves a series of enzymatic reactions mediated by three enzymes: E1 (ubiquitin-activating enzyme), E2 (ubiquitin-conjugating enzyme), and E3 (ubiquitin ligase), with E3s being responsible for determining substrate specificity (2, 3, 4). The poly-ubiquitin modification can be removed to achieve protein homeostasis by deubiquitinases (DUBs), a class of specialized proteases that removes polyubiquitin chains from protein substrates (5, 6, 7, 8, 9).

RAS proteins have been shown to undergo ubiquitin-mediated regulations. Three E3 ubiquitin ligases have been reported to facilitate RAS polyubiquitination and subsequent degradation, β-TRCP for HRAS (10); NEDD4-1 (11) and WDR76 (12) for all three RAS homologs. An additional E3 ligase, leucine-zipper–like transcriptional regulator 1 (LZTR1), was found responsible for monoubiquitination and diubiquitination of RAS proteins. This monoubiquitin/diubiquitin modification interferes with the proper localization of RAS GTPases to the plasma membrane and disrupts RAS signaling (13, 14). Recent evidence also suggests that LZTR1 may polyubiquitinate RAS for degradation (15, 16), which is regulated by GSK and implicated in neoplastic transformation (16). Genetic studies in fruit fly as well as in mice indicate that the preferred substrate of LZTR1 is actually RIT1, a noncanonical RAS protein (17). Both *LZTR1* and *RIT1* are mutated in Noonan syndrome, a condition characterized by RAS overactivation (18). These results reveal that the ubiquitin modification of RAS plays important roles in regulating RAS signaling.

Whether there is redundancy among the identified RAS ubiquitin ligases or whether they are utilized differentially in varying conditions or cell types remains elusive. Furthermore, there should be DUBs which could act on RAS so that their ubiquitin modification process is kept in check to allow precise control over their abundance and activity. Thus, we launched an siRNA screen against the majority of DUBs in human genome to find those important in regulating, specifically, KRAS protein abundance. The screen identified the DUB USP25. We show here that KRAS is a substrate of USP25. Depleting *USP25* expression in cancer cells increases ubiquitination and proteasomal degradation of KRAS, leading to the suppression of its downstream MAP kinase pathway and cell proliferation. We further show that the inhibitor of USP25 could destabilize KRAS in cancer cells and was efficacious in blocking tumor xenograft growth in mice. These results indicate that the DUB USP25 is an indirect and yet effective target for suppressing KRAS, and its inhibition can potentially aid in the treatment of tumors driven by KRAS activating mutations.

## Results

### Identification of DUBs that control KRAS protein expression

To identify DUBs that are required to maintain KRAS protein abundance in cancer cells, we performed an siRNA screen in human colon cancer cell line HCT116 which carries a G13D mutation in *KRAS*. A commercial DUB siRNA library designed against 90 DUBs with each DUB targeted by 3 siRNAs (in most cases) was utilized. We transfected each individual siRNA into HCT116, harvested the cells 48 h post the transfection, and examined KRAS levels in these cells *via* immunoblotting (Fig. S1*A*). The expression levels of KRAS were quantitated and normalized to that in control cells and plotted (Fig. S1*B*), and the knockdown efficiency of each siRNA in the library was also assessed with quantitative polymerase chain reaction (qPCR) analysis of the respective DUB (Fig. S1*C*). When at least two out of three siRNAs against a DUB could reduce KRAS expression levels more than 50%, the DUB was considered a potential regulator of KRAS expression. Based on these two criteria, USP13, USP25, USP30, OTUD4, and USP45 were chosen and those initially effective siRNAs were screened for a second time (Fig. S1*C*). Only USP13 and USP25 passed the secondary screen (Fig. S1*D*). Since the changes in KRAS protein levels could also come from changes in its mRNA levels, we analyzed *KRAS* mRNA expression *via* qPCR (Fig. S1*E*). It turned out that the depletion of *USP13* expression decreased *KRAS* mRNA levels, suggesting that *USP13* likely controls *KRAS* transcription instead of being a DUB for KRAS. On the other hand, the depletion of *USP25* expression did not alter *KRAS* mRNA levels, making USP25 a likely KRAS DUB.

**Figure 1:**
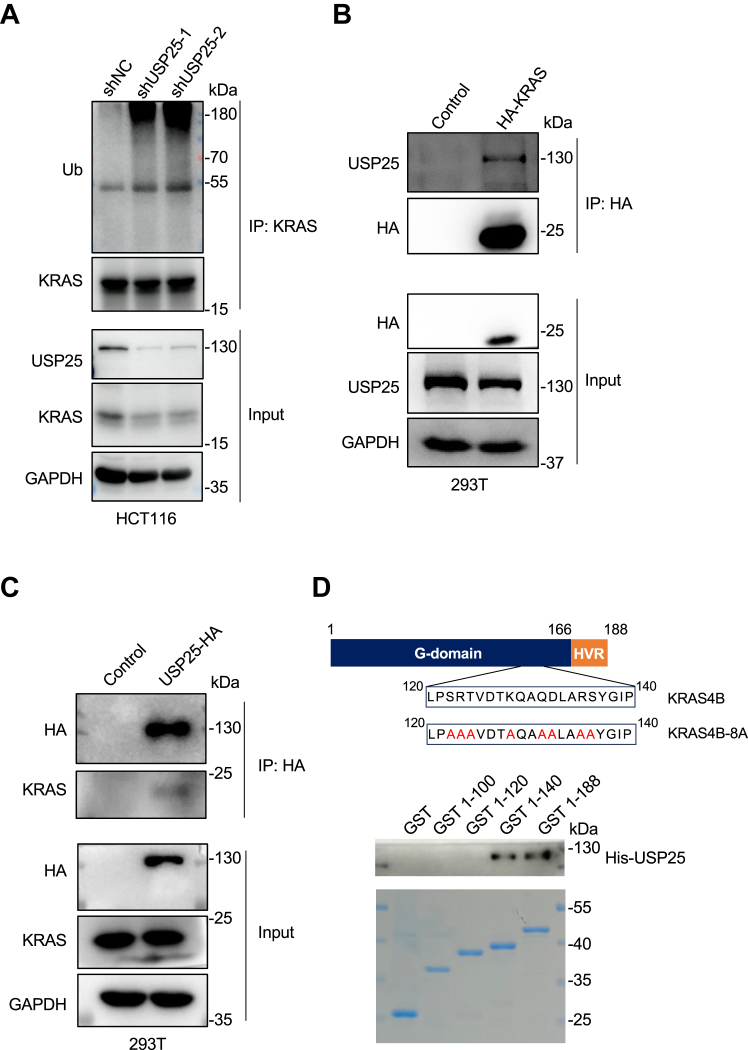

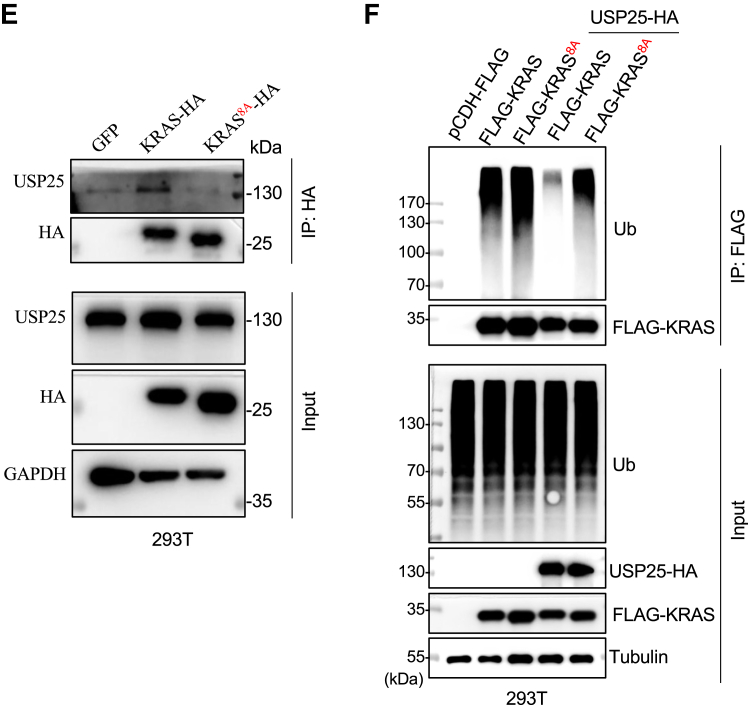
**USP25 is a deubiquitinase for KRAS.***A*, USP25 deubiquitinates KRAS. KRAS ubiquitination levels were assessed *via* immunoprecipitation and Western blotting following the knockdown of *USP25* with two independent shRNAs in HCT116 cells. The amounts of total proteins from *USP25-*depleted cells were adjusted for immunoprecipitation to reflect the fact that these cells now contained less KRAS protein. *B*, coimmunoprecipitation analysis of the interaction between HA-tagged KRAS and the endogenous USP25 in HEK293T cells. *C*, coimmunoprecipitation analysis of the interaction between HA-tagged USP25 and the endogenous KRAS in HEK293T cells. *D*, GST pull-down assay with GST-KRAS (full length and truncations) and His-USP25. A diagram of KRAS protein is presented. His-USP25 was detected with Western blotting (*upper panel*). The GST and GST fusion proteins were separated in an SDS-PAGE gel and visualized with Coomassie blue staining (*lower panel*). *E*, coimmunoprecipitation analysis of the interaction between HA-tagged KRAS (WT and the 8A mutant, *D*) and the endogenous USP25 in HEK293T cells. *F*, analysis of the ubiquitination levels in KRAS (WT and the 8A mutant, *D*) in 293T cells with or without USP25 overexpression. GST, glutathione-*S*-transferase.

As a DUB of KRAS, we expect that depleting *USP25* would increase the levels of KRAS ubiquitination. Indeed, the levels of ubiquitination on KRAS increased in HCT116 cells (Fig. 1*A*) as well as in HT29 cells (Fig. S1*F*) when the expression of *USP25* was compromised. As expected, USP25 could interact with KRAS (Fig. 1*B*) and *vice versa* (Fig. 1*C*). To map the site in KRAS that interacts with USP25, we expressed and purified a series of truncated glutathione-*S*-transferase (GST)-KRAS4B proteins to pulldown His-USP25 (Fig. 1*D*). This GST pull-down assay implicated a 20-residue region located between residues 120 and 140 responsible for the interaction with USP25. Mutating 8 charged or polar residues to alanine in this region (KRAS-8A, Fig. 1*D*) abolished the interaction with USP25 in HCT116 cells (Fig. 1*E*). As a result, unlike its WT counterpart, the ubiquitination levels on 8A mutant were no longer affected by the overexpression of *USP25* (Fig. 1*F*). Further, this 8A mutant is still functional (Fig. S2*A*), indicating that it can fold correctly and the effect on interaction with USP25 is not due to misfolding.

It is known that *KRAS* is expressed as two splicing variants, *KRAS4B* (the main form, usually referred as *KRAS*) and *KRAS4A* (19). Since the antibodies used above (and through the rest of the work) could not differentiate the two isoforms, we wondered if USP25 regulated only a specific KRAS isoform. To that end, using KRAS4A and B-specific antibodies, we found that both KRAS4A and KRAS4B require the function of USP25 to maintain their expression (Fig. S2*B*). Furthermore, there are two other RAS proteins, HRAS and NRAS. We wondered if they are also regulated by USP25. Indeed, depleting the expression of the DUB results in the downregulation of both HRAS and NRAS (Fig. S2*C*), suggesting USP25 likely function as a DUB for all three RAS proteins.

Taken together, these data demonstrate that compromising the function of the DUB *USP25* can downregulate KRAS expression and suppress KRAS signaling.

### Mapping the ubiquitination sites in KRAS

**Figure 2:**
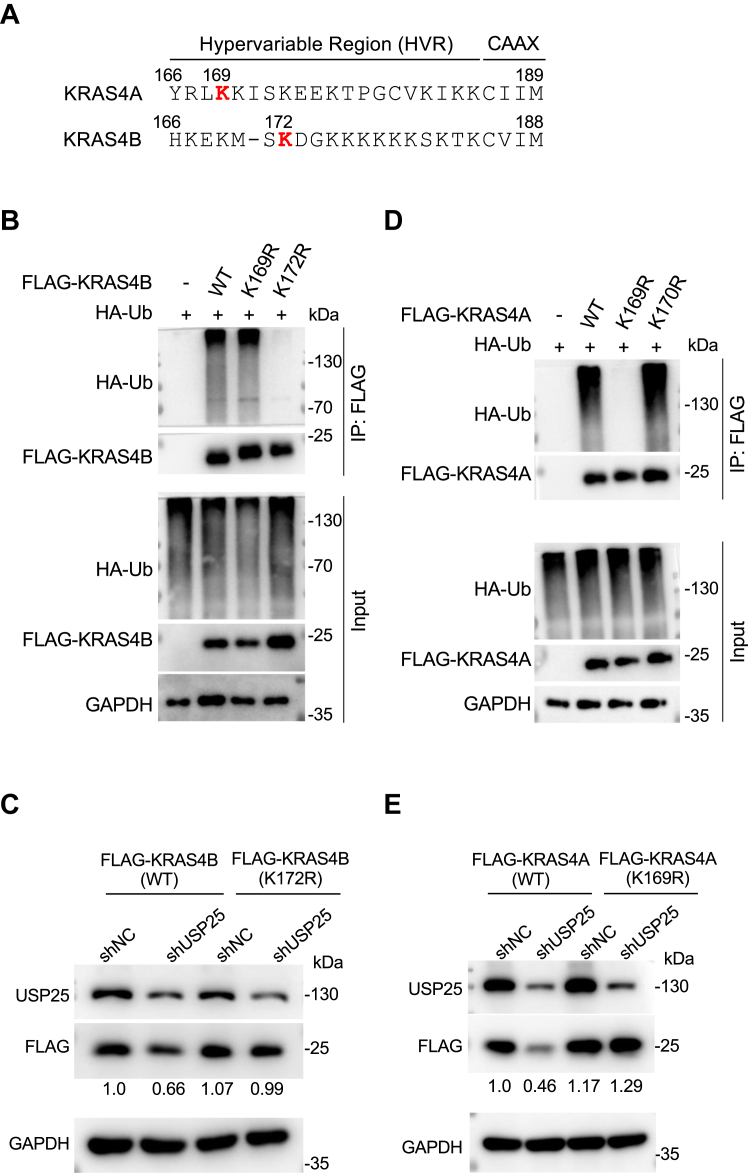
**Identification of the ubiquitination sites in KRAS.***A*, the hypervariable regions of KRAS4A and KRAS4B. The *red highlighted lysine residues* are responsible for KRAS ubiquitination. *B*, analysis of the ubiquitination levels in KRAS4B mutants. Flag-tagged WT KRAS4B, KRAS4B^K169R^, or KRAS4B^K172R^ were expressed in HEK293T cells and immunoprecipitated for Western blotting analysis of ubiquitination levels. *C*, KRAS4B^K172R^ no longer requires *USP25* for stable expression. HEK293T cells were first transfected with Flag-tagged KRAS4B WT or K172R mutant and divided into two parts which were then infected with lentiviruses carrying a shUSP25-expressing cassette or shNC control cassette. Flag-tagged KRAS4B protein levels were analyzed with Western blotting. *D*, analysis of ubiquitination levels in KRAS4A mutants. Flag-tagged WT KRAS4A, KRAS4A^K169R^, or KRAS4A^K172R^ were expressed in HEK293T cells and immunoprecipitated for Western blotting analysis of ubiquitination levels. *E*, KRAS4A^K169R^ no longer requires *USP25* for stable expression. HEK293T cells were first transfected with Flag-tagged KRAS4A WT or K169R mutant and divided into two parts which were then infected with lentiviruses carrying a shUSP25-expressing cassette or shNC control cassette. Flag-tagged KRAS4A protein levels were analyzed with Western blotting.

Despite several reports of E3 ubiquitin ligases of RAS proteins (10, 11, 12, 15, 16), no lysine residues have been identified that are ubiquitinated and mediate the proteasomal degradation of RAS proteins. Four lysine residues, K104, K117, K128, and K147, were shown to be monoubiquitinated to regulate KRAS activation and signaling (20, 21, 22, 23, 24). Indeed, mutating these residues to arginine did not affect polyubiquitination of KRAS (Fig. S3*A*). It was shown previously that RasG, a KRAS ortholog in *dictyostelium*, was subjected to ubiquitin-mediated proteasomal degradation and 4 lysine residues in the C-terminal hypervariable region (HVR) were shown to be critical for this process (25). Interestingly, human KRAS4B expressed in *dictyostelium* was also subjected to ubiquitination and degradation (25). Given the similarities in the HVR between RasG and KRAS4B, we suspected that the lysine residue(s) responsible for KRAS4B ubiquitination and subsequent degradation might reside in the HVR (Fig. 2*A*). To that end, we first replaced all but 2 lysine residues in the region, generating a 9KR mutant of KRAS4B (Fig. S3*B*). This mutant lost almost all ubiquitination in 293T cells (Fig. S3*C*). From there, we generated a 4KR mutant (Fig. S3*B*) which was unable to be polyubiquitinated as the 9KR mutant (Fig. S3*C*), suggesting that the bulk of the ubiquitination modification occur on some or all of these 4 lysine residues. Therefore, we replaced these 4 lysine residues with arginine one at a time and each replacement was assessed for its ability to be ubiquitinated. Satisfactorily, K172 was found to be responsible for all polyubiquitination on KRAS4B (Figs. 2, *A* and *B* and S3*D*), and KRAS4B^K172R^ is no longer dependent on *USP25* for stable expression (Fig. 2*C*).

Similarly, we mapped the ubiquitination sites in KRAS4A. First, we replaced all 7 lysine residues in the HVR to arginine to generate a 7KR mutant (Fig. S3*E*). The ubiquitination on KRAS4A^7KR^ was nearly abolished (Fig. S3*F*), indicating that like KRAS4B, the ubiquitination of KRAS4A also occurs in the HVR. We subsequently generated a 4KR mutant by replacing K169, K170, K173, and K176 with arginine (Fig. S3*E*). Again, this 4KR mutant seemed unable to be ubiquitinated (Fig. S3*F*), suggesting that the ubiquitination takes place on one or more of these 4 residues. We then tested the possibility that the ubiquitination on KRAS4A takes place on one lysine residue, much like KRAS4B as shown above, by generating each individual arginine replacement. Indeed, this is the case. K169 turned out to be responsible for the ubiquitination of KRAS4A (Figs. 2, *A* and *D* and S3*G*) and mutating this residue to arginine similarly stabilized KRAS4A when *USP25* was depleted (Fig. 2*E*).

To determine whether the activating mutations of KRAS would interfere with its ubiquitination, we generated a double mutant KRAS-4B in which G13 was changed to D and K172 to R. While KRAS^G13D^ could be ubiquitinated as its WT counterpart, the double mutant (KRAS^G13D/K172R^) showed much reduced ubiquitination (Fig. S3*H*).

### USP25 is necessary for KRAS signaling and promotes cancer cell proliferation

**Figure 3:**
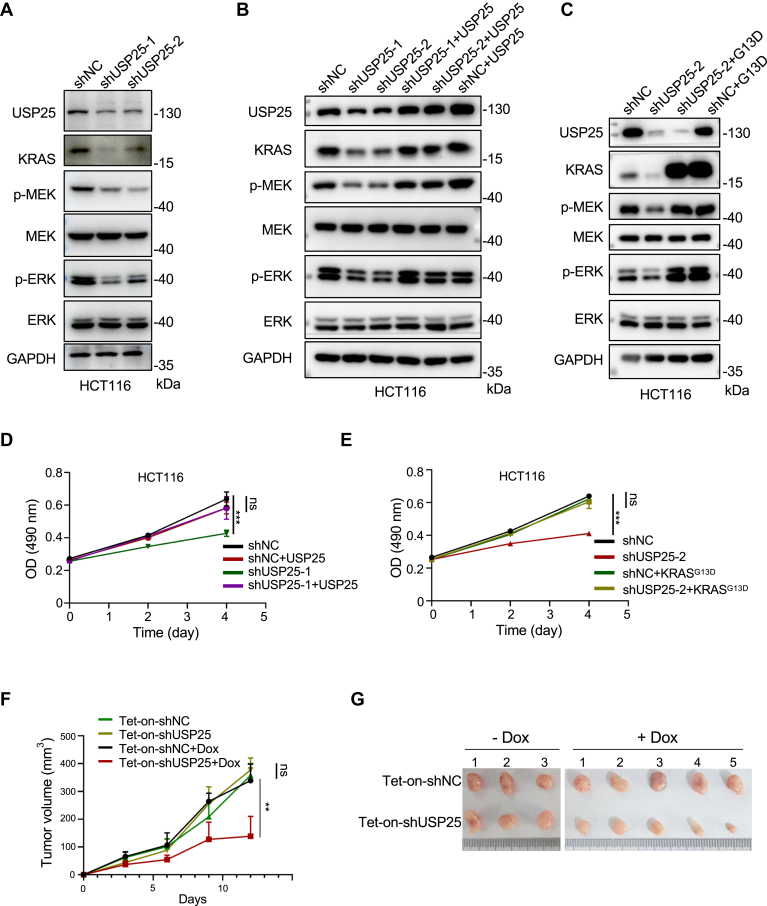

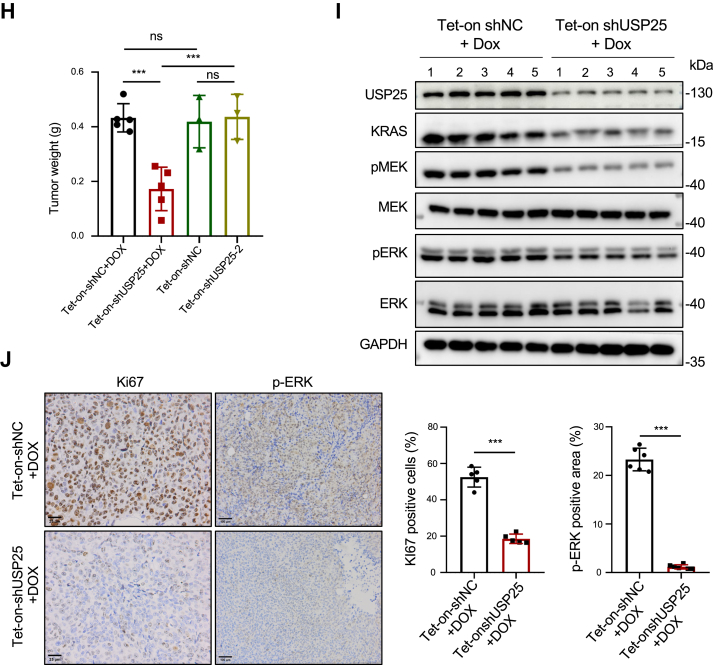
**The proliferation of tumor cells requires *USP25*.***A*, Western blotting analysis of the expression levels of KRAS and its downstream signaling pathway proteins following *USP25* knockdown in HCT116 cells. *B*, Western blotting analysis of the expression levels of KRAS and its downstream signaling pathway proteins following *USP25* knockdown with shUSP25-1 and shUSP25-2 and re-expression of *USP25* in HCT116 cells. *C*, Western blotting analysis of the expression levels of KRAS and its downstream signaling pathway proteins following *USP25* knockdown with shUSP25-2 and exogenous expression of *KRAS*^*G13D*^ in HCT116 cells. *D*, growth curve analysis of HCT116 cells with *USP25* expression depleted (with shUSP25-1) or depleted plus re-expression of *USP2*5. *E*, growth curve analysis of HCT116 cells with *USP25* expression depleted (with shUSP25-2) or depleted plus exogenous expression of *KRAS*^*G13D*^. *F*, the growth of the xenograft tumors derived from HCT116 cells carrying doxycycline-inducible shUSP25 expression cassette (Tet-on-shUSP25) or a negative control (Tet-on-shNC). *G*, photographic representation of tumors excised from the nude mice. *H*, the weight of tumors in (*C*). *I*, Western blotting analysis for KRAS and its downstream signaling proteins in the tumor samples. *J*, immunohistochemical (IHC) analysis of the expression of proliferation marker Ki67 and phosphor-ERK (p-ERK) in tumor tissue sections along with quantitation of the IHC staining. One section from each one of the 5 tumor samples was stained and quantitated. Data are presented as mean ± SD. ∗∗*p* < 0.01 and ∗∗∗*p* < 0.001.

To determine if the downregulation of KRAS resulted from depleting the expression of *USP25* impacts KRAS downstream signaling, we examined the phosphorylation status of MEK as well as that of ERK. As shown in Figure 3*A*, the depletion of *USP25* decreased the phosphorylation levels of both MEK and ERK, which could be rescued by re-expressing *USP25* (Fig. 3*B*) or be prevented by exogenously expressed *KRAS*^*G13D*^ (Fig. 3*C*). The downregulation of KRAS signaling was also observable in other cancer cell lines carrying different *KRAS* mutations, including the pancreatic cancer cell line Capan-2 (*KRAS*^*G12V*^) (Fig. S4*A*), and the lung cancer cell lines H23 (*KRAS*^*G12C*^) and A549 (*KRAS*^*G12S*^) (Fig. S4*B*). It is known that KRAS can activate AKT signaling (26). We therefore wondered if *USP25* also plays a role in that regard. Indeed, depleting *USP25* led to reduced levels of active (phosphorylated) AKT, which could be rescued by re-expressing *KRAS* (Fig. S4*C*).

Given the well-documented importance of KRAS signaling in cell proliferation, it is expected that compromising *USP25* would hamper cell proliferation. Indeed, depleting *USP25* expression with two shRNAs (carrying the sequences identical to the two siRNAs identified in the initial screen, Fig. S1) suppressed the growth of HCT116 cells (Figs. 3*D* and S5*A*), and re-expressing *USP25* (Fig. 3*B*) could bring the growth rates back (Figs. 3*D* and S5*A*). In HCT116, this slowdown of growth was not because the cells were dying through apoptosis (Fig. S5*C*), but because they were slow to enter S phase (Fig. S5*D*). The requirement of *USP25* for optimal growth was also evident in other cancer cells (Fig. S5*B*). Importantly, expressing *KRAS*^*G13D*^ in *USP25-*depleted HCT116 cells (Fig. 3*C*) canceled the growth-inhibiting effect of *USP25* depletion (Fig. 3*E*). Together, these data strongly support the notion that *USP25* promotes cancer cell growth, through (at least partly) stabilizing KRAS expression.

To demonstrate the role of *USP25* in tumor growth *in vivo,* we established doxycycline (Dox)-inducible shUSP25 (and shNC as control) expressing HCT116 cells. The shNC and shUSP25 cells were inoculated in nude mice side by side. When the xenografts reached a size of ∼50 mm^3^, the drinking water for the mice was switched to that containing Dox. The progression of these xenograft tumors was assessed by measuring the volume of the tumors at 3-day intervals. As shown in Figure 3*F*, inducing shUSP25 expression suppressed tumor growth, while the growth rates between shNC and shUSP25 tumors were similar if Dox was not administered. The xenograft tumors were harvested by day 12 for analyses. Consistent with their slow growth rates, shUSP25 tumors were much smaller than the controls (Fig. 3, *G* and *H*). Immunoblotting of the tumor protein extracts showed that in the *USP25-*depleted tumors KRAS protein levels as well as the phosphorylation levels of MEK and ERK were all downregulated (Fig. 3*I*). Further immunohistochemical (IHC) staining demonstrated much reduced proliferation (Ki67 positive) and phospho-ERK expression in shUSP25-expressing tumors (Fig. 3*I*). Taken together, these results demonstrate that *USP25* is crucial for KRAS signaling and tumor growth.

### Pharmacological inhibition of USP25 can temper KRAS signaling

**Figure 4:**
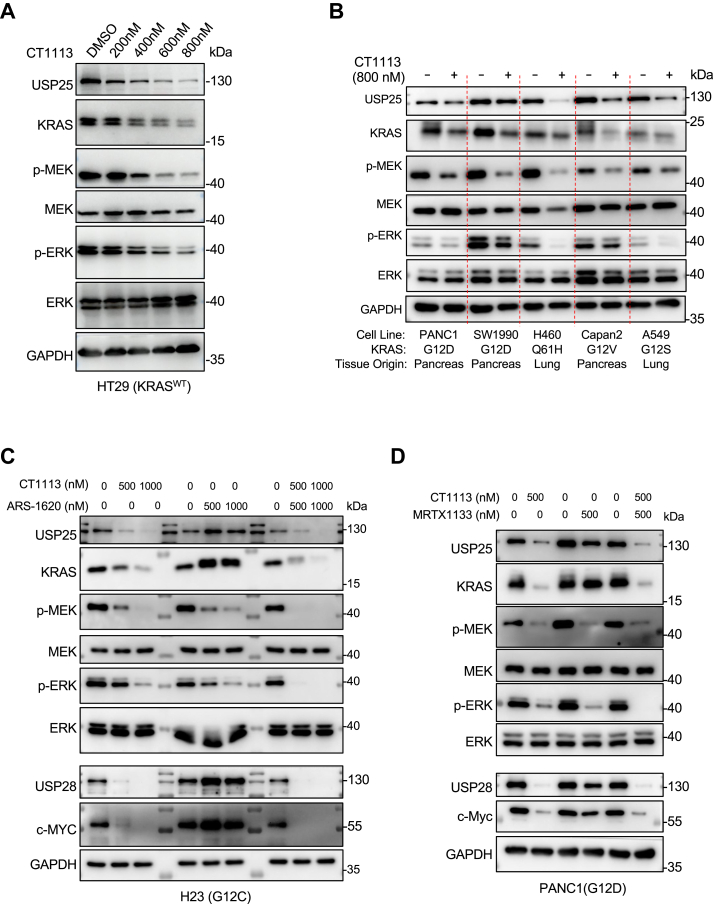
**Pharmacological inhibition of USP25 downregulates KRAS expression and its downstream signaling.***A*, analysis of KRAS signaling pathway proteins with Western blotting analysis in HT29 cells treated with different concentrations of CT1113 for 72 h. *B*, Western blotting analysis of KRAS signaling pathway proteins across multiple cell lines treated with 800 nM CT1113 for 72 h. *C*, examination of KRAS signaling pathway proteins with Western blotting analysis in H23 cells treated with CT1113 and ARS-1620 individually or in combination for 72 h. Since CT1113 also inhibits USP28, the expression levels of USP28 and its substrate c-MYC were also examined. *D*, examination of KRAS signaling pathway proteins with Western blotting analysis in PANC1 cells treated with CT1113 and MRTX1133 individually or in combination for 72 h. The expression levels of USP28 and its substrate c-MYC were also examined.

We recently developed a strong inhibitor of USP25 (and its homolog USP28), CT1113 (27). To determine if pharmacological inhibition of USP25 is effective in reducing the expression levels of KRAS protein and in tempering KRAS signaling, we treated HT29 colon cancer cells (which do not harbor mutations in *KRAS*) with different concentrations of CT1113 (Fig. 4*A*). As expected, KRAS levels as well as the phosphorylation levels of MEK and ERK decreased in a dose-dependent manner. Moreover, inhibiting USP25 could temper KRAS signaling in pancreatic, lung, and colon cancer cells carrying various activating mutations in *KRAS* and for one of the mutants, *KRAS*^*Q61H*^, no direct inhibitors are available (Fig. 4*B*). The ubiquitin level on KRAS increased as expected when USP25 was inhibited (Fig. S6*A*).

Inhibiting USP25 caused its own downregulation in many cell lines (Fig. 4). This can be explained by USP25 being its own DUB. The downregulation also depends on cell lines. Apparently, it is not as substantial in PANC1 and SW1990 as in H460, Capan2, and A549 (Fig. 4*B*), which might be caused by different USP25 E3 activities in different cells. In addition, since the inhibitor inhibits both USP25 and USP28, we wanted to know if USP28 plays any role in regulating KRAS ubiquitination. We depleted *USP28* expressing in HCT116 and HT29 cells with 3 different shRNAs, but detected no effects on KRAS expression levels (Fig. S6, *B* and *C*). This result indicates that USP28 contribute little to KRAS ubiquitination homeostasis.

There have been mutant KRAS inhibitors reported. We therefore compared CT1113 with KRAS^G12C^ inhibitor ARS-1620 (28) in H23 lung cancer cells which carry *KRAS*^*G12C*^, and with KRAS^G12D^ inhibitor MRTX1133 (29, 30) in PANC1 pancreatic cancer cells which carry *KRAS*^*G12D*^. As shown in Figure 4, *C* and *D*, CT1113 is as effective as ARS-1620 or MRTX1133 in blocking KRAS signaling, indicating that reducing the expression levels of KRAS protein can produce similar inhibitory effects on KRAS signaling as direct inhibition of the GTPase. Furthermore, stronger inhibitory effects against KRAS signaling could be achieved when CT1113 and the KRAS inhibitors were combined (Fig. 4, *C* and *D*), this additive effect could be seen at even low concentrations of the inhibitors (Fig. S6, *D* and *E*). As an inhibitor of USP28, CT1113 treatment also downregulated c-MYC levels as we reported previously (27), which the KRAS inhibitors are incapable of (Fig. 4, *C* and *D*).

**Figure 5:**
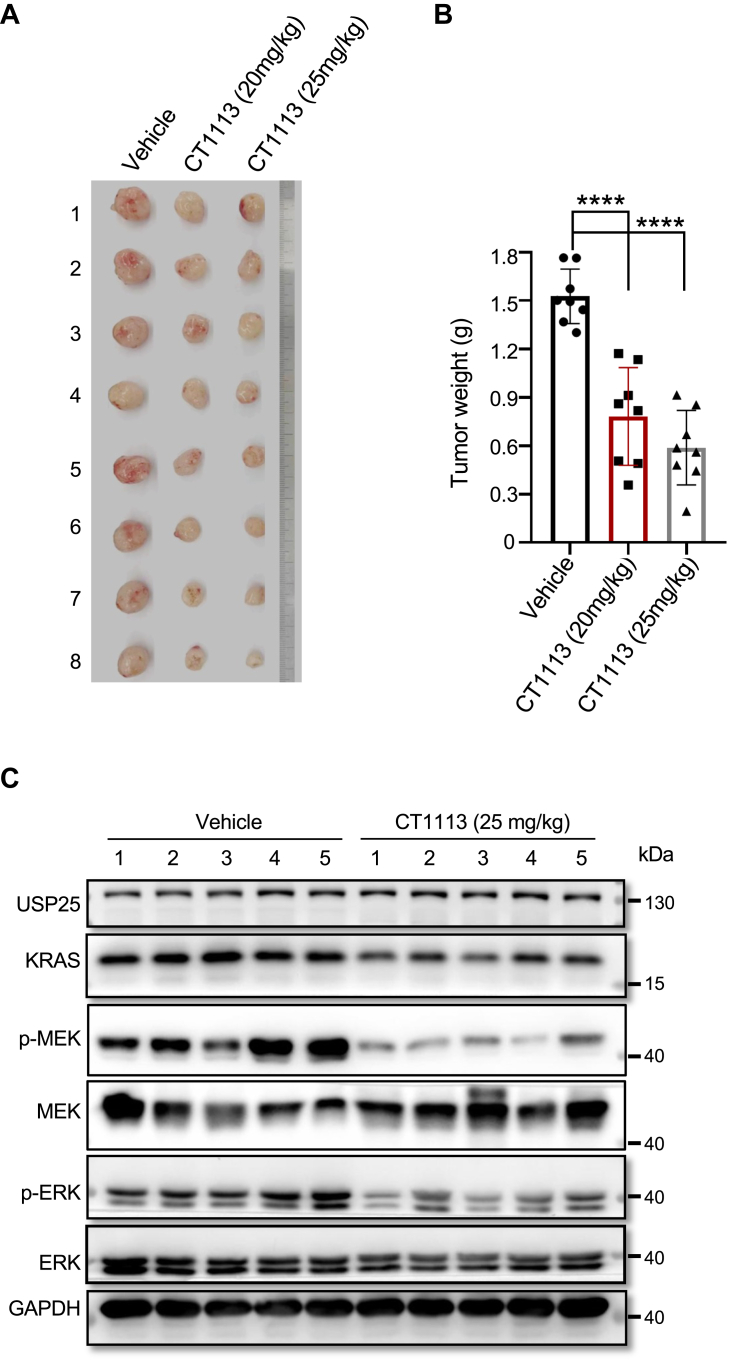
**Pharmacological inhibition of USP25 suppresses tumor growth.***A*, photographs of the xenograft tumors derived from human pancreatic cancer cell line SW1990. 1 × 10^6^ SW1990 cells were inoculated in each nude mouse. When the xenografts grew to palpable sizes, the mice were given CT1113 orally (20 or 25 mg/kg body weight) twice a day for 2 weeks. *B*, the weight of the tumors in (*A*). *C*, Western blotting analysis of KRAS signaling pathway proteins in vehicle control and CT1113 (25 mg/kg) treated tumors. Data are presented as mean ± SD. ∗∗∗∗*p* < 0.0001.

Next, we applied CT1113 *in vivo* to determine if it is effective in blocking KRAS signaling in xenograft tumors. We inoculated nude mice with SW1990 pancreatic cancer cells (which are *KRAS*^*G12C*^) and started to treat these mice with CT1113 (20 or 25 mg/kg, bid.) when the xenografts had grown to palpable sizes. The tumors were harvested for analysis after 2 weeks of CT1113 treatment. As expected, CT1113 treatment suppressed the growth of SW1990 xenograft tumors (Fig. 5, *A* and *B*). The expression levels of KRAS as well as the phosphorylation levels of MEK and ERK were all downregulated in the tumors treated with CT1113 (Fig. 5*C*). Similar results were obtained in a patient-derived colon cancer xenograft model (CoY1607, from OncoCare Biotech) (Fig. S7). This model contains WT *KRAS*, but CT1113 was similarly effective in blocking KRAS signaling. In our previous report (27), CT1113 was shown to be effective in inhibiting the growth of xenograft tumors derived from HCT116 cells (which are *KRAS*^*G13D*^) and the compound is tolerable in mice.

### USP25 expression correlates with RAS expression and unfavorable prognosis

**Figure 6:**
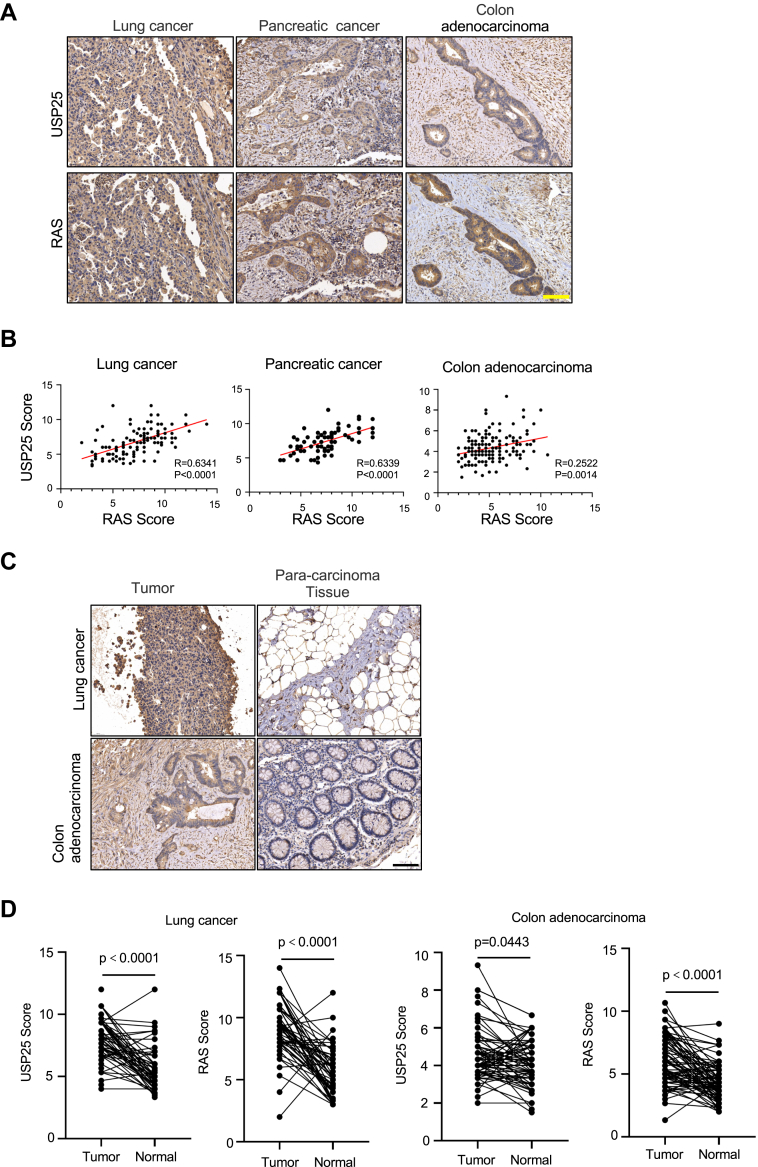
**The expression of USP25 correlates with RAS in human cancer.***A*, representative immunohistochemical staining images from tissue microarrays (TMAs) for USP25 and RAS proteins. The human lung and colon adenocarcinoma TMAs consist of 80 pairs of cancerous and para-cancerous tissues. The human pancreatic cancer TMA consists of cancerous tissues only. The scale bar represents 100 μm. *B*, the correlation between USP25 and RAS expression in human cancers. The expression scores of the two proteins in TMAs were plotted against each other. *C*, enhanced expression of USP25 and RAS in human cancer specimens relative to adjacent nontumor tissues. Shown are representative images of IHC staining of USP25 and RAS in cancerous and para-cancerous tissues of the lung cancer and colon adenocarcinoma samples. No para-cancerous tissues are available in the pancreatic cancer samples for comparison. *D*, pair-wise comparison of USP25 and RAS expression in cancerous and para-cancerous tissues from (*C*). IHC, immunohistochemical.

Having shown the importance of USP25 in maintaining KRAS expression and signaling, we next sought to determine if the expression of the DUB and KRAS are correlated in human tumors. We stained human cancer tissue arrays for USP25 and RAS (Figs. 6*A* and S8*A*). The anti-RAS antibodies were unable to distinguish K-, H-, and N-RAS and were reported previously suitable for IHC staining (31, 32). The expression of USP25 and RAS were scored and plotted (Fig. 6*B*). Strong correlations between USP25 expression and that of RAS were detected across lung, pancreatic, and colon cancers (Fig. 6*B*). In addition, USP25 expression in tumor tissues of lung and colon cancer samples is notably elevated compared to the adjacent noncancerous tissues (Fig. 6, *C* and *D*). Moreover, the expression of *USP25* is inversely correlated with patient survival in lung, colon, and pancreatic cancers (Fig. S8*B*).

## Discussion

**Figure 7:**
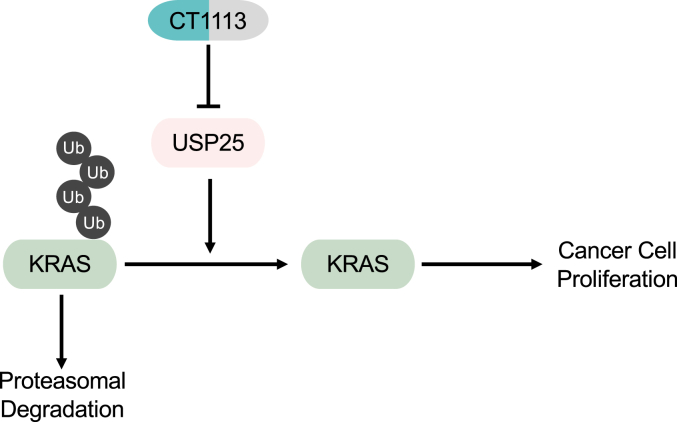
**A schematic illustration of the regulation of KRAS by USP25.** The expression of KRAS proteins depends on USP25. Compromising USP25 function can lead to suppression of KRAS.

The ubiquitin-proteasome system, through the action of E3 ligases and the counteraction of DUBs, maintains protein homeostasis. Perturbing the function of E3s or DUBs can disrupt this homeostasis, which has been exploited by cancer cells. For example, the E3 ligase *FBW7* is frequently mutated in human cancer due to its ability to target oncoproteins such as MYC for proteasomal degradation (33, 34). The RAS family of small GTPases are also subjected to this homeostatic regulation by a number of E3 ligases (10, 11, 12, 15, 16) and by the DUB USP25 identified here. We show here that disrupting this homeostasis through compromising USP25 is a viable approach to suppress KRAS. Genetic depletion of *USP25* expression or pharmacological inhibition of the DUB causes decreases in the expression levels of KRAS (WT or mutants) and in the strength of KRAS downstream signaling, resulting in reduced rates of cancer cell proliferation (Fig. 7).

The development and clinical application of KRAS^G12C^ inhibitors have effectively overcome the previously perceived undruggable nature of KRAS (35, 36). Nonetheless, the emergence of drug resistance is unavoidable during the course of treatment with KRAS inhibitors (37). A side-by-side comparison of USP25 inhibitor (CT1113) with KRAS^G12C^ inhibitor (ARS-1620) (Fig. 4*C*), or with KRAS^G12D^ inhibitor (MRTX1133) (Fig. 4*D*), demonstrated that the inhibition of USP25 is as effective as the direct inhibition of KRAS. These findings suggest that USP25 inhibitors may serve as viable alternatives to KRAS inhibitors for suppressing KRAS, particularly in cases where resistance to KRAS inhibitors has manifested. In addition, those KRAS mutants which are nontargetable currently, such as Q61X, could be suppressed by compromising *USP25* function (Fig. 4*B*), adding more value to USP25 inhibitors as alternative KRAS blockers.

*USP25* was initially found to be involved in immune signaling through regulating ubiquitin modifications of TRAF proteins (38, 39). Since then, a number of proteins have been shown to require USP25 for their homeostasis, including Tankyrase (40), BCR-ABL (41), EGFR (42), HIF-1α (43), and KEAP1 (44), which are all implicated in tumorigenesis one way or another. *USP25* has a close homolog, *USP28*, and the two DUBs share 64% similarity. Due to this high degree of similarity, CT1113 can inhibit both with similar potency (27). *USP28* is more implicated in cancer than *US25*, as it has a larger number of onco-proteins on its client list, including prominent ones like c-MYC (45), MYCN (46), LSD1 (47), *etc.* Now, with the identification of KRAS here as dependent on USP25 for its expression, the USP25/28 dual are gaining more importance as targets for cancer therapy, since their inhibition can now simultaneously suppress both c-MYC and KRAS (Fig. 4, *C* and *D*), the two most prominent onco-drivers.

There was a recent report that USP7 could deubiquitinate KRAS and maintain its expression in small cell lung cancer cells (48). In our DUB library screen, the siRNAs against *USP7* did cause its mRNA levels to about 50% of the control (Fig. S1*C*), but we did not observe appreciable downregulation of KRAS protein levels (Fig. S1, *A* and *B*), likely because there were still sufficient levels of USP7 protein translated from the remaining mRNA that were able to prevent excess ubiquitination of KRAS. It is not unusual that more than one DUBs work on the same target just like there are more than one E3s that can ubiquitinate a target protein. Alternatively, USP7’s action on KRAS might be cell context-dependent. In any case, this reflects the complexity of protein homeostasis control.

## Experimental procedures

### Cell culture

HEK293T, HCT116, HT29, NCI-H23, A549, Capan-2, SW1990, H460, PANC1, and LLC cells were purchased from American Type Culture Collection. The cells were maintained in either Dulbecco's modified Eagle's medium, RPMI-1640, McCoy's 5A supplemented with 10% fetal bovine serum, 1% Penicillin/Streptomycin at 37 °C with 5% CO_2._

### siRNA library screening

An siRNA library targeting 84 human DUBs, and a control siRNA were purchased from RiboBio. Each DUB is targeted with three siRNAs. HCT116 cells were reverse-transfected with 100 nM siRNAs using Lipofectamine RNAiMAX (Invitrogen) for 48 h. The cells were then harvested and lysed for analysis. A secondary screening was performed similarly with the identified siRNAs in the primary screen.

### Cell proliferation and apoptosis assay

The cells were seeded in a 96-well plate and incubated for 3 to 5 days. After the incubation period, the culture medium was replaced, and 100 μl of fresh medium along with 20 μl of 3-(4,5-Dimethylthiazol-2-yl)-5-(3-carboxymethoxyphenyl)-2-(4-sulfophenyl)-2H-tetrazolium (Promega) solution was added. The cells were then incubated for an additional 2 h. Finally, the absorbance at 490 nm as an indicator of the number of live cells was measured using a microplate reader.

For cell cycle analysis, the cells were trypsinized, collected by centrifugation, washed once in cold PBS, and fixed in 70% ice-cold alcohol over 1 h. The cells were then spun down, washed with cold PBS, and incubated in PBS containing propidium iodide (PI, 50 μg/ml) and RNase A (50 μg/ml) for 30 min at room temperature. The PI-stained single cell suspension was analyzed on a BD LSRFortessa SORP Flow Cytometer (BD Biosciences). ModFit LT software (Verity Software House, https://www.vsh.com) was used to analyze the DNA patterns and cell cycle stages. For apoptosis assay, the cells were trypsinized, collected by centrifugation, washed once with cold PBS, and stained with annexin V-FITC (Abcam) and PI for 15 ∼ 30 min at room temperature. The cells were then analyzed with a flow cytometer.

### RNA isolation and RT-PCR

Total RNA was extracted from cells using TRIzol (Sigma-Aldrich) according to the manufacturer’s instructions. RNA was reverse-transcribed into complementary DNA using a reverse transcription kit (AG). Complementary DNA was used for RT-qPCR reaction (AG). The primers used are listed inTable S1.

### Western blotting analysis

Cells or tissues were lysed with RIPA buffer (Applygen) supplemented with protease inhibitors and phosphatase inhibitors (Roche), and the lysates were centrifuged at 10,000 g for 10 min at 4 °C to remove insoluble debris. The protein concentration of the resulting lysates was determined using a bicinchoninic acid assay kit (Beyotime). Equal amounts of total protein were boiled for 10 min in 5× SDS loading buffer, separated on SDS-polyacrylamide gel, and transferred onto nitrocellulose membranes. The membranes were blocked with 5% nonfat dry milk in Tris-buffered saline with Tween 20: 20 mM TrisHCl, pH8.0/150 mM NaCl/0.1% Tween 20 (TBST) for 1 h at room temperature to block nonspecific binding. Then, the membranes were incubated with primary antibodies overnight at 4 °C. After three washes with TBST, the membranes were incubated with horseradish peroxidase–conjugated secondary antibodies for 1 h at room temperature. The membranes were washed three times and visualized using SuperSignal West Pico Chemiluminescent substrate (Thermo Fisher Scientific). Expression of GAPDH was commonly used as a loading control. The antibodies used are listed in Table S2.

### Ubiquitination assay

HEK293T and HCT116 cells were transfected with HA-KRAS and other relevant plasmids. Forty eight hours of after the transfection, the cells were treated with 10 μM MG132 for 6 to 8 h, and then harvested and lysed in NETN buffer (pH 8.0 Tris–HCl, 100 mM NaCl, 1 mM EDTA, 0.5% Nonidet P-40) containing 1% SDS and 1% sodium deoxycholate. The lysates were vortexed vigorously for 15 to 30 min and boiled for 10 min. After that, 10 times the volume of NETN buffer was added to reduce the SDS content to 0.1%, and the lysates were centrifuged at 10,000 g at room temperature to remove cell debris. The resulting cell lysates were incubated with appropriate antibody-conjugated beads followed by immunoprecipitation procedures.

### GST pull-down assay

KRAS4B fused to GST and USP25 fused to 6XHis were expressed in *Escherichia coli* and purified with glutathione-agarose and nickel beads, respectively. His-USP25 was eluted out of the beads with PBS containing 250 mM imidazole and 500 mM NaCl. His-USP25 was incubated with GST-bound USP25 on the beads at 4 °C for 4 h. The beads were then washed five times with GST buffer (1 mM EDTA, pH 8.0 Tris–HCl, 200 mM NaCl, and 1% Triton X-100), and the bound proteins were eluted from the beads using SDS gel sample buffer, separated in an SDS-PAGE gel, and analyzed with Western blotting using specific antibodies.

### Immunoprecipitation assay

The cells were lysed in NETN buffer for 30 min and centrifuged to remove cell debris. The resulting supernatant was the cell lysate. The cell lysates were then incubated with Sepharose beads (such as Flag M2 beads, Sigma) or protein A/G-conjugated Sepharose beads, which were conjugated with specific antibodies. The incubation was carried out at room temperature for 1 h or overnight at 4 °C. After the incubation, the beads were washed at least 3 times with NETN buffer. The proteins bound to the beads were eluted by boiling in SDS-gel loading buffer and analyzed by Western blotting.

### Plasmids and lentiviruses

Plasmids utilized in this work were generated through conventional cloning techniques. Specifically, the shRNAs were constructed in pLKO.1 vector using the following sequences: shNC (5′-TTCTCCGAACGTGTCACGT-3′), shUSP25-1 (5′-GCGTGAGCTGAGGTATCTATT-3′), and shUSP25-2 (5′-GCACTTCTCCTGTTGACGATA-3′). Human *USP25* and *KRAS4A/B* coding sequences were cloned into the pCDH vector with a Flag or HA tag. Lentiviruses for shRNA or gene expressing were produced in 293T cells using standard packaging plasmids and procedures. Lentiviral infection of cells was conducted using standard protocols, and the infected cells were selected with 2 μg/ml puromycin (Beyotime) for 2 days to establish stable cell lines.

### Animal experiments

All animal experiments were approved by the Animal Care and Use Committee of the First Affiliated Hospital of Zhejiang University. Nude mice were purchased from Hangzhou Ziyuan Experimental Animal Technology Co, Ltd.

To generate cell-derived xenograft tumors, Tet-on-shNC or Tet-on-shUSP25 HCT116 cells were mixed at a 1:1 ratio (volume) with matrigel (BD Biosciences) and injected subcutaneously into 5- to 6-week-old BALB/c nude mice. After successful engraftment (when the xenograft tumors reached a size of ∼50 mm^3^), the mice were given 0.2% Dox and 5% sucrose-containing drinking water and sacrificed 12 days later. The tumors were dissected out and cut into 3 equal parts: one for protein/RNA extraction, one for histology, and one for frozen storage.

To test the efficacy of CT1113 *in vivo*, an established patient-derived colon cancer xenograft model (CoY1607) obtained from OncoCare Biotech, Inc. was used with the following procedure. A CoY1607 tumor was dissected out of its host, washed twice in PBS to remove necrotic tissue, and cut into small pieces of equal sizes and placed on ice. The tumor cubes were inoculated subcutaneously into nude mice within an hour of preparation. CT1113 treatment (20 mg/kg body weight, twice a day through an oral gavage) was started once the tumor mass became palpable. At the end of treatment, the tumor-bearing animals were sacrificed, and the tumor mass was dissected out for analyses. In addition, xenograft tumors derived from SW1990 cells were used for testing CT1113. For that, 1 × 10^6^ cells were mixed at a 1:1 volume ratio with matrigel (BD Biosciences) and inoculated subcutaneously BALB/c nude mice. When the xenograft tumors reached approximately 50 mm^3^ in size, the mice were randomly divided into three groups, one group for vehicle control, and the other two for the administration of CT1113 at 20 or 25 mg/kg body weight, twice a day *via* oral gavage. CT1113 treatment lasted 27 days. At the conclusion of the treatment period, the tumors were excised and divided into three equal parts: one for protein/RNA extraction, one for histological examination, and one for cryogenic preservation.

### IHC staining

Tumor issues were fixed in 10% formaldehyde at 4 ^o^C overnight. Tissue processing, sectioning, and histological and IHC staining were performed by Servicebio. Each tumor sample was sectioned and the sections with largest tissue area were selected for IHC staining. For Ki67 scoring, three fields of view were randomly selected per section, and the ratio of Ki67-positive cells to the total number of cells within the field of view is calculated to determine the Ki67 positivity rate. The average of these rates is taken. For p-ERK scoring, again three fields of view are selected, and the ratio of the positive-staining area to the total area within these fields is calculated as the p-ERK score, with the average of these scores being determined.

Human lung cancer, colon adenocarcinoma, and pancreatic cancer tissue arrays were purchased from Xiangyou Tech, and the provider also performed IHC staining of USP25 and RAS. The IHC staining strength was unbiasedly assessed using a composite positive scoring system, which is the product of staining intensity and the percentage of positive cells. The IHC staining intensity is graded on a scale from 0 to 4 as follows: 0: no observable staining; 1: very weak staining, barely perceptible; 2: weak staining, light yet clearly visible; 3: moderate staining, strong and distinct; 4: strong staining, very intense, and prominent (Fig. S8*A*). The percentage of positive cells was scored as follows: 0: 0 to 5% positive cells; 1: 6 to 25% positive cells; 2: 26 to 50% positive cells; 3: 51 to 75% positive cells; and 4: >75% positive cells. Scoring was independently conducted by three pathologists, with the final score being the average of their assessments.

### Statistical analysis

Correlation analysis was performed using the Spearman correlation test to assess the relationship between variables. For paired samples, the Wilcoxon matched-pairs signed rank test was utilized. The significance between two groups was assessed using an unpaired two-tailed Student's *t* test. Statistical analyses were conducted using GraphPad Prism 9.0 (https://www.graphpad.com). A *p* value of less than 0.05 was considered statistically significant (∗*p* < 0.05; ∗∗*p* < 0.01; ∗∗∗*p* < 0.001; and ∗∗∗∗*p* < 0.0001).

## Data availability

All data are contained in this article.

## Supporting information

This article contains supporting information.

## Conflict of interest

The authors declare that they have no conflicts of interest with the contents of this article.
