# Supplementary material for: USP25 maintains KRAS expression and inhibiting the deubiquitinase suppresses KRAS signaling in human cancer
Source: J Biol Chem. 2025 Jun 3;301(7):110337. doi: 10.1016/j.jbc.2025.110337 (PMC12269508; doi:10.1016/j.jbc.2025.110337)
Supplement: Supplemental Tables [file mmc1.pdf]

Supplemental Table 1. Primers used for RT-PCR

Name	Sequence
USP25-F	GCACCAGCAGACGTTTTTGAA
USP 25-R	AGCATTCTTCGCAGTAAGGAAA
USP13-F	CAGATGCGACCTGCGAGAAAA
USP13-R	TGGGTAGCCCATGTCTCTGTA
KRAS-F	GAGTACAGTGCAATGAGGGAC
KRAS-R	CCTGAGCCTGTTTTGTGTCTAC
GAPDH-F	AGGTGAAGGTCGGAGTCAAC
GAPDH-R	CGCTCCTGGAAGATGGTGAT

Supplemental Table 2. Antibody List

Antibody	Supplier	Cat No.	Working Condition
KRAS	Proteintech	Cat No. 12063-1-AP	1:1000 for WB
KRAS4B	Proteintech	Cat No. 16155-1-AP	1:1000 for WB
KRAS4A	Proteintech	Cat No. 16156-1-AP	1:1000 for WB
GAPDH	Proteintech	Cat No. 60004-1-Ig	1:5000 for WB
USP25	ABclonal	A23431	1:1000 for WB
HIS	Proteintech	Cat No. 66005-1-Ig	1:1000 for WB
ERK	CST	#4695	1:1000 for WB
MEK	CST	#4694	1:1000 for WB
pMEK	CST	#3958	1:1000 for WB
USP28	Proteintech	17707-1-AP	1:1000 for WB
HA	Sigma	H6908	1:2500 for WB
flag	Proteintech	80010-1-RR	1:5000 for WB
USP25	Sigma	HPA024142	1:200 for IHC
RAS	CST	#67648	1:500 for IHC
p-ERK	CST	#4370	1:1000 for WB<br>1:200 for IHC
KI67	Servicebio	GB121141	1:400 for IHC
