# Supplementary material for: USP25 maintains KRAS expression and inhibiting the deubiquitinase suppresses KRAS signaling in human cancer
Source: J Biol Chem. 2025 Jun 3;301(7):110337. doi: 10.1016/j.jbc.2025.110337 (PMC12269508; doi:10.1016/j.jbc.2025.110337)
Supplement: Supplemental Figure Legend [file mmc2.docx]

**Supplemental Figure Legend**

**Figure S1.** **Identification of USP25 as a putative deubiquitinating enzyme for KRAS.**

**A.** Analysis of KRAS protein levels following the knockdown of individual DUBs in HCT116 cells. siRNAs were transfected into HCT116 cells, and the endogenous levels of KRAS were analyzed by Western blotting 48 hrs after the transfection.

**B.** Quantification and normalization of KRAS expression in (A). The intensity of protein bands in (A) was measured with ImageJ. In each batch of transfection of siRNAs and western blotting, the KRAS expression level (KRAS intensity over that of GAPDH) in siNC cells was set to 1. Red dashed lines mark the cut-off (50% KRAS expression relative to the expression level in siNC).

**C.** Quantitation of DUB expression with qPCR in HCT116 cells transfected with the DUB siRNA library. Each siRNA in the library was transfected into 3 different wells of cells and the cells were harvested 48 h later for total RNA extraction and qPCR analysis. The mRNA expression of each DUB after the siRNA transfection was normalized to the cells transfected with control siRNA.

**D.** Validation of candidate KRAS-regulating siRNAs in HCT116 cells. The cells were transfected with the same siRNAs found in **A.**, targeting USP13, USP25, USP30, OTUD4, and USP45. The endogenous expression levels of KRAS were measured with western blotting assay.

**E.** Assessment of KRAS mRNA levels following the knockdown of USP13 and USP25 in HCT116 cells. Data are presented as mean ± SD. ***, p < 0.001; ns, no significance.

**F.** Ubiquitination assay of KRAS in HT29 cells depleted of *USP25* expression.

**Figure S2. Regulation of RAS proteins by USP25**

**A.** USP25-interaction deficient KRAS mutant (KRAS^8A^) is catalytically functional.

**B.** The depletion of *USP25* expression decreases the expression of both KRAS4A and KRAS4B, the two isoforms of *KRAS*.

**C.** Western blotting analysis of all three RAS proteins in HCT116 cell depleted of *USP25.*

**Figure S3. Identification of ubiquitination sites in KRAS isoforms.**

**A.** Analysis of the ubiquitination levels in KRAS4B mutants. Flag-tagged wild-type KRAS4B (WT), KRAS4BK^104R^, KRAS4B^K117KR^, KRAS4BK^128R^, or KRAS4B^K147KR^ were co-expressed with HA-ubiquitin in 293T cells, immunoprecipitated and analyzed.

**B.** A schematic representation of the hypervariable region of KRAS4B and two K-to-R replacement mutants, 9KR and 4KR. Potential ubiquitination sites were highlighted with red.

**C.** Analysis of the ubiquitination levels in KRAS4B mutants. Flag-tagged wild-type KRAS4B (WT), KRAS4B^4KR^, or KRAS4B^9KR^ were expressed in HEK293T cells and immunoprecipitated for western blotting analysis of the ubiquitination levels.

**D.** Analysis of the ubiquitination levels in KRAS4B mutants. Flag-tagged wild-type KRAS4B (WT), KRAS4BK^177R^, KRAS4B^K175KR^, KRAS4BK^172R^, or KRAS4B^K169KR^ were co-expressed with HA-ubiquitin in 293T cells, immunoprecipitated and analyzed.

**E.** A schematic representation of the hypervariable region of KRAS4A and two K-to-R replacement mutants, 7KR and 4KR. Potential ubiquitination sites were highlighted with red.

**F.** Analysis of ubiquitination levels in KRAS4A mutants. Flag-tagged wild-type KRAS4A (WT), KRAS4A^4KR^, or KRAS4A^7KR^ were expressed in HEK293T cells and immunoprecipitated for western blotting analysis of ubiquitination levels.

**G.** Analysis of the ubiquitination levels in KRAS4A mutants. Flag-tagged wild-type KRAS4A(WT), KRAS4A^K176R^, KRAS4A^K173R^, KRAS4A^K170R^, or KRAS4A^K169R^ were co-expressed with HA-ubiquitin in 293T cells, immunoprecipitated and analyzed.

**H.** Analysis of the ubiquitination of the activating KRAS mutant, KRAS^G13D^. Flag-tagged wild-type KRAS-4B (KRAS), G13D mutant (KRA^G13D^), K172R mutant (KRAS^K172K^), or the double mutant (KRAS^G13D/K172R^) were co-expressed with HA-ubiquitin in 293T cells, immunoprecipitated and analyzed.

**Figure S4. USP25 maintains KRAS expression and signaling in various cancer cell lines.**

**A.** Western blotting analysis of the expression levels of KRAS and its downstream signaling pathway proteins following *USP25* knockdown in Capan-2 cells.

**B.** Western blot analysis of the expression levels of KRAS and its downstream signaling pathway proteins following *USP25* knockdown in NCI-H23 and A549 cells.

**C.** Analysis of phospho-AKT levels in HCT116 cells depleted of *USP25* with or without *KRAS4B* re-expression.

**Figure S5. Knockdown of USP25 inhibits tumor cell proliferation.**

**A.** Growth curve analysis of HCT116 cells with *USP25* expression depleted (with shUSP25-2) or depleted plus re-expression of *USP25*.

**B.** Growth curve analysis of SW1990, Capan-2, NCI-H23, and A549 cells with *USP25* expression depleted (with shUSP25-1 or -2).

**C.** FACS analysis of apoptosis in HCT116 cells depleted of *USP25.*

**D.** FACS analysis of the cell cycle in HCT116 cells depleted of *USP25.* The distribution of the cells in cycle phases was plotted.

Data are presented as mean ± SD. ***, p < 0.001; ns, no significance.

**Figure S6. The effect of USP25 inhibition on KRAS expression signaling.**

**A.** Analysis of KRAS ubiquitination levels. KRAS-HA was expressed in 293T cells, immunoprecipitated and analyzed. The cells were treated with 800 nM CT1113 for 72 hrs before harvesting.

**B, C.** Western blotting analysis of KRAS in *USP28-*depleted HCT116 (**B**) and H29 (**C**) cells.

**D.** Examination of KRAS signaling pathway proteins with western blotting analysis in H23 cells treated with CT1113 and ARS-1620 individually or in combination for 72 hours.

**E.** Examination of KRAS signaling pathway proteins with western blotting analysis in PANC1 cells treated with CT1113 and MRTX1133 individually or in combination for 72 hours.

**Figure S7. USP25 inhibitor CT1113 could suppress xenograft tumor growth.**

A) The growth of CoY1607 patient-derived colon cancer xenograft tumors in nude mice treated with or without CT1113 (20 mg/kg, bid).

B) Photographs of the tumors in (A).

C) The weight of the tumors in (A).

D) Western blotting analysis of KRAS signaling pathway proteins in vehicle control and CT1113 treated tumors.

Data are presented as mean ± SD. ****, p < 0.0001.

**Figure S8. USP25 expression correlates with prognosis**

**A.** Scoring of IHC staining intensity. 0: no observable staining. 1: very weak staining, barely perceptible. 2: weak staining, light yet clearly visible. 3: moderate staining, strong and distinct. 4: strong staining, very intense and prominent. Scale bar, 100 µm.

**B.** The KM-plot of *USP25* expression and cancer patient survival. The data were obtained from GEPIA2.
