# Supplementary figures and images for: USP25 maintains KRAS expression and inhibiting the deubiquitinase suppresses KRAS signaling in human cancer
Source: J Biol Chem. 2025 Jun 3;301(7):110337. doi: 10.1016/j.jbc.2025.110337 (PMC12269508; doi:10.1016/j.jbc.2025.110337)

A

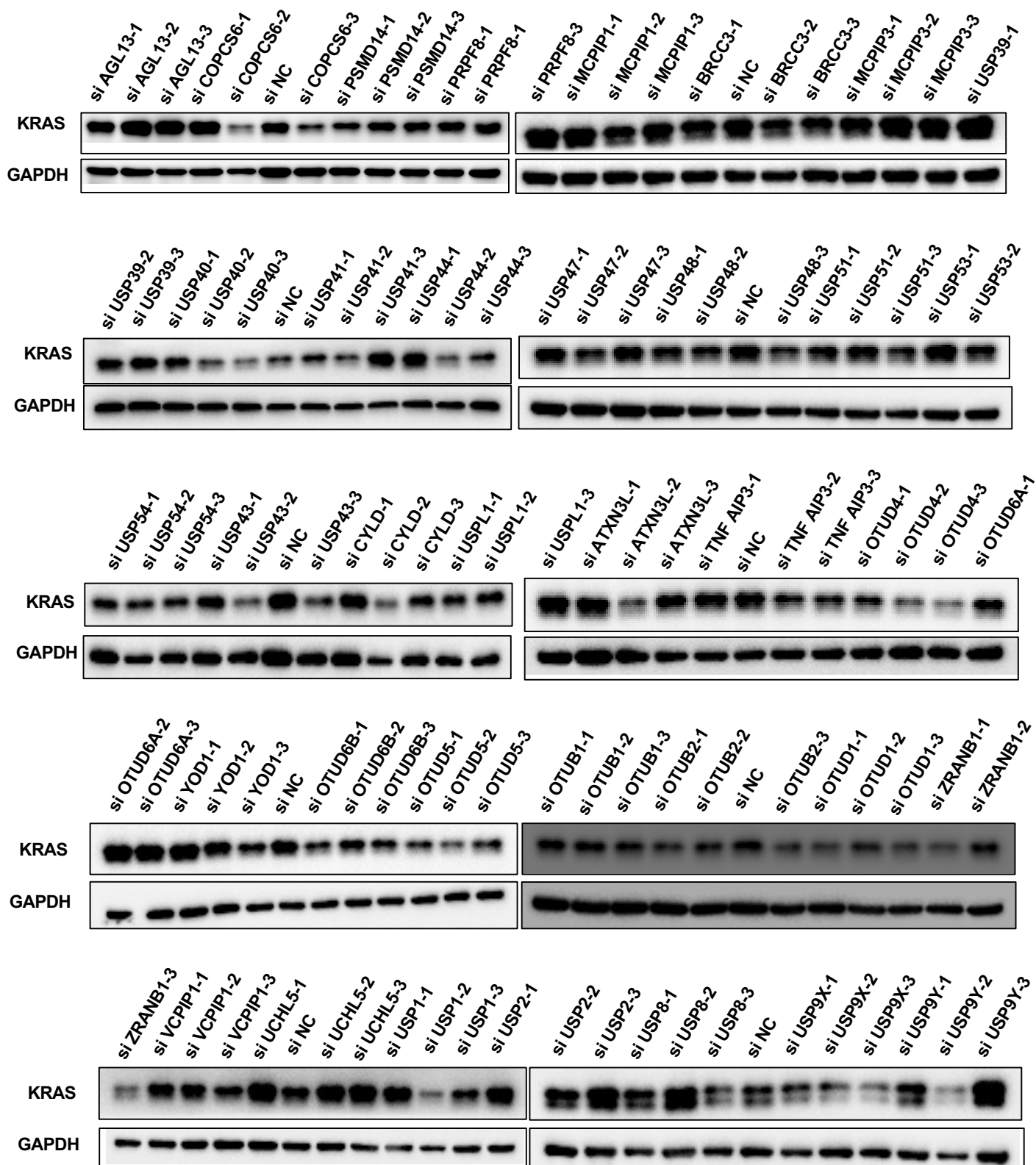

Figure S1

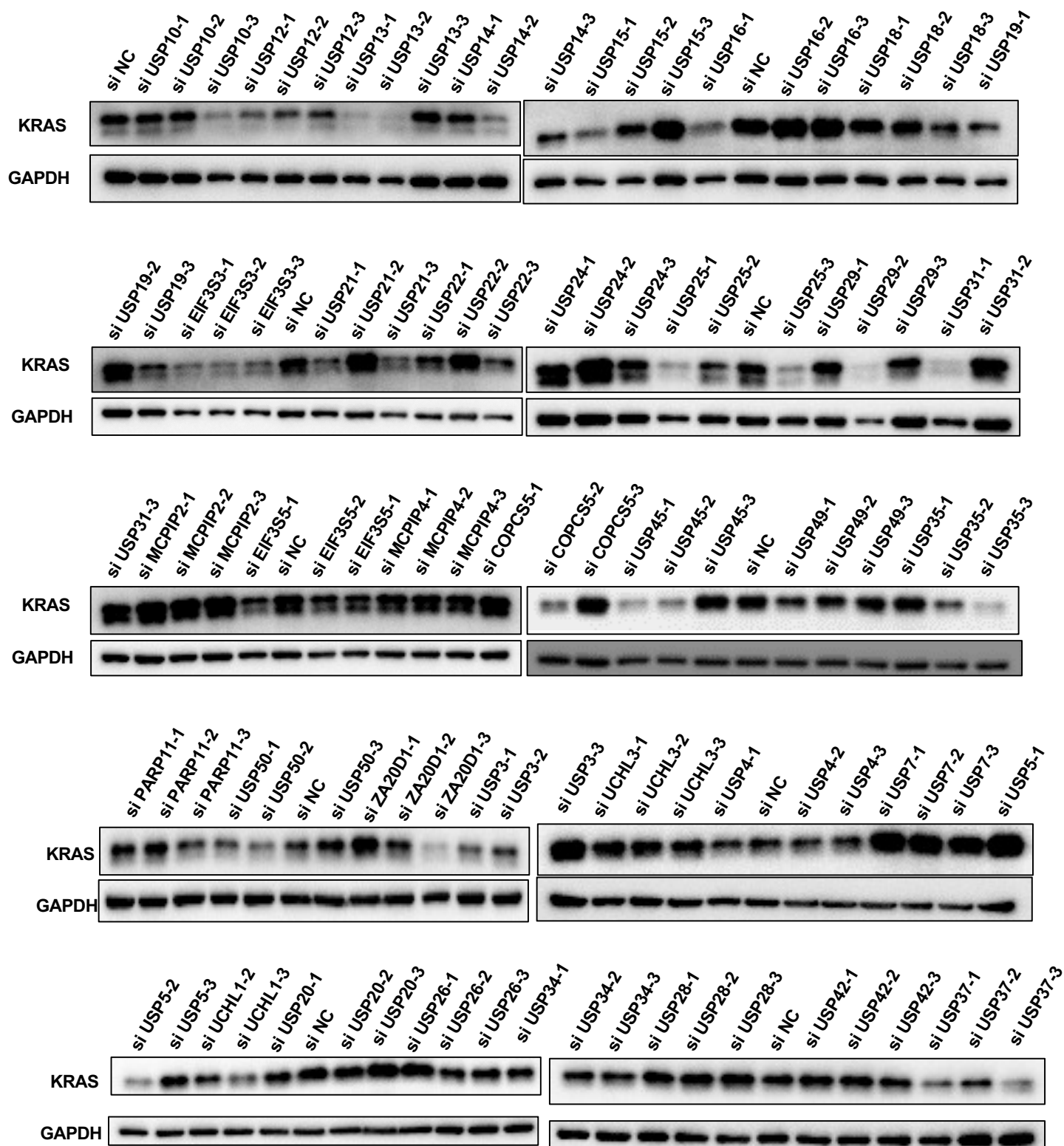

Figure S1

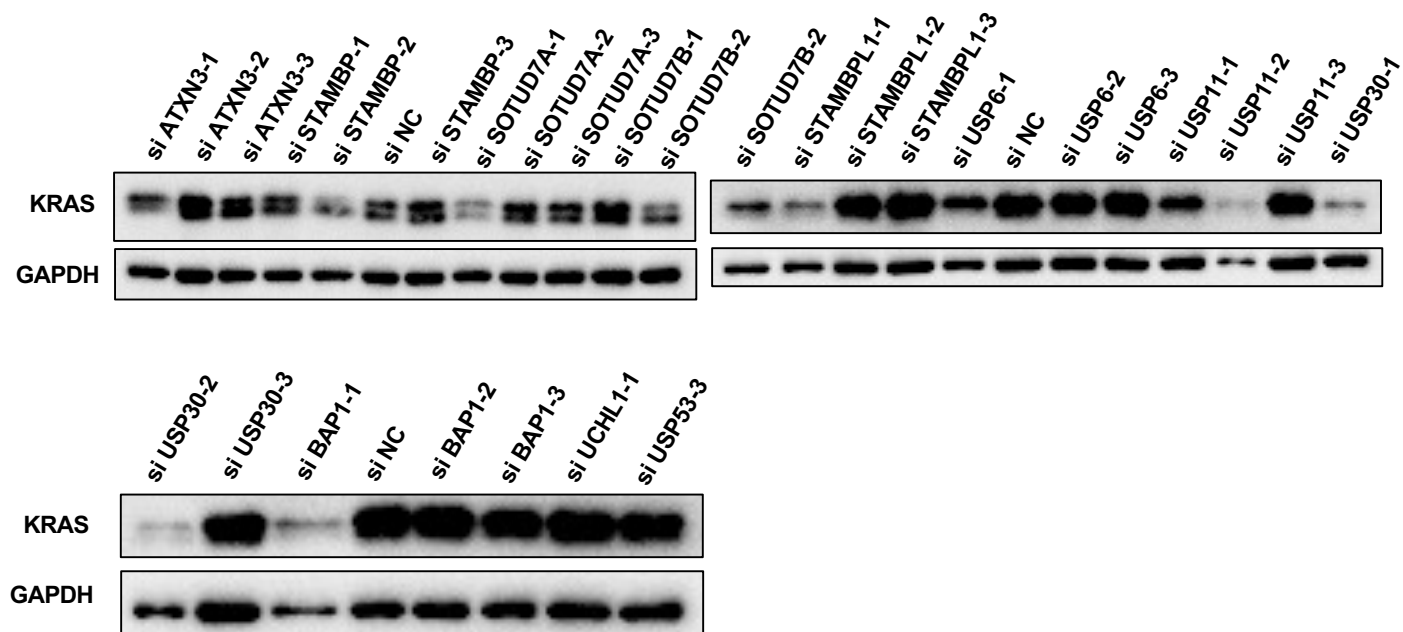

Figure S1

**B**

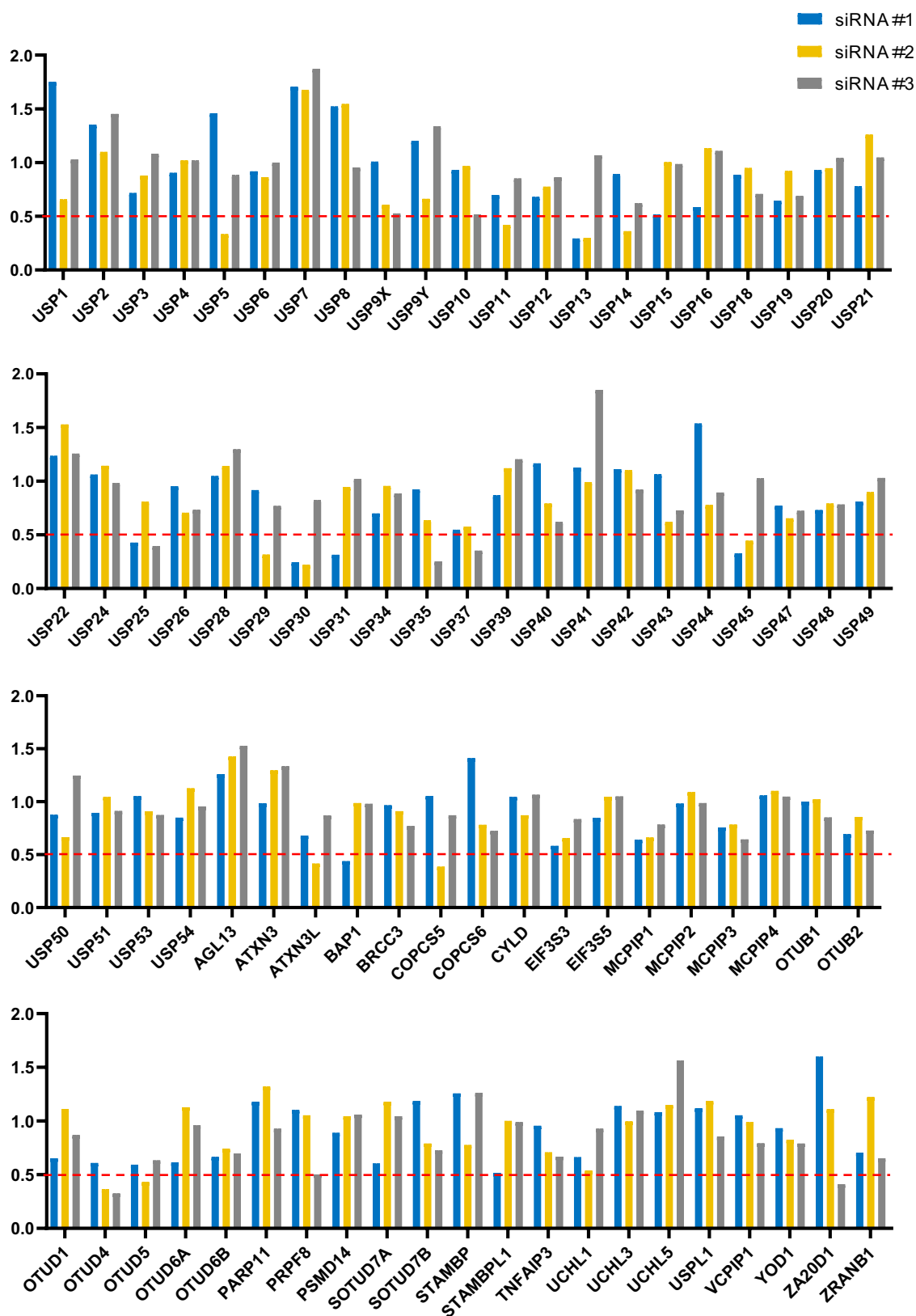

**Figure S1**

C

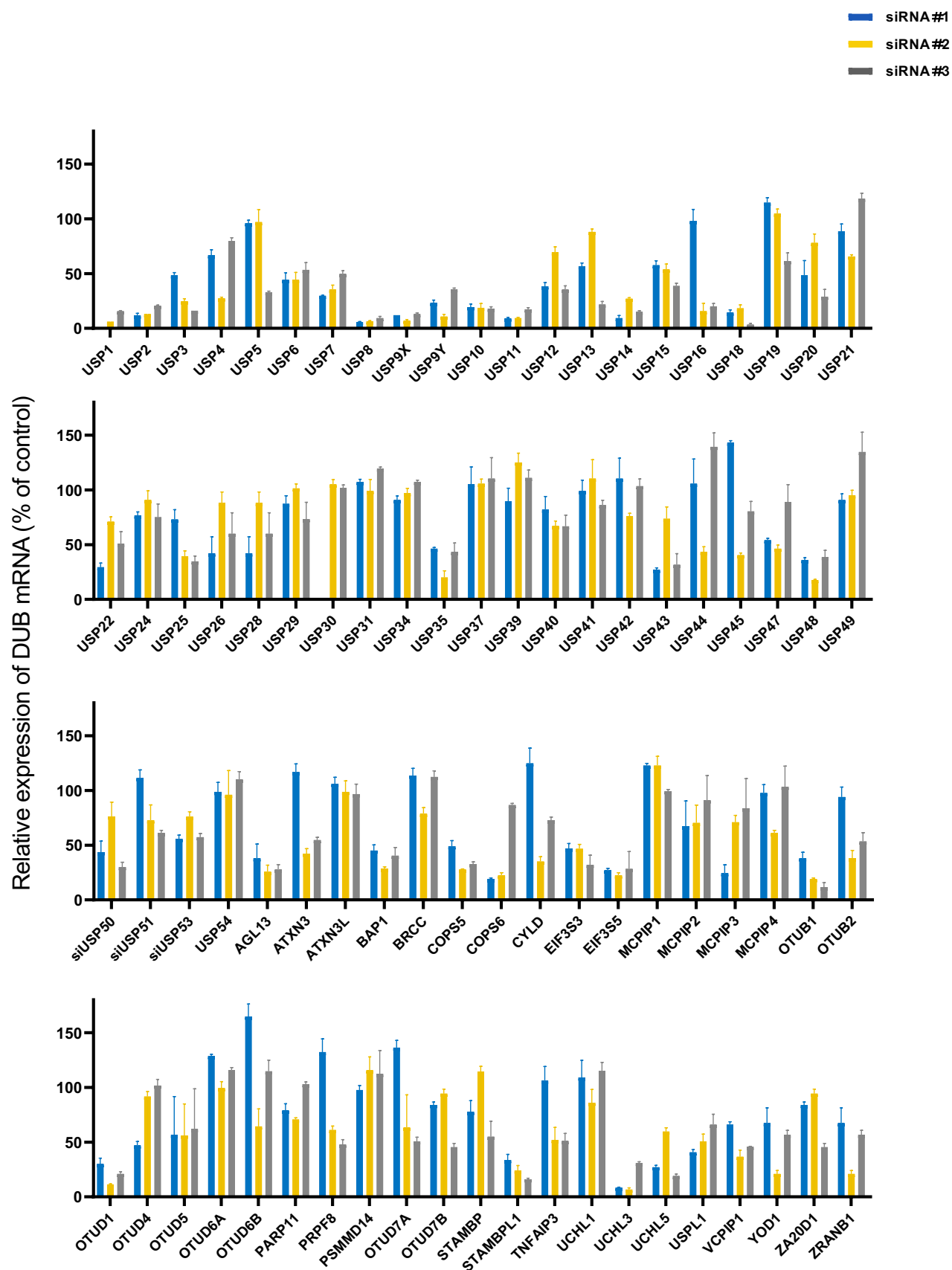

Figure S1

**D**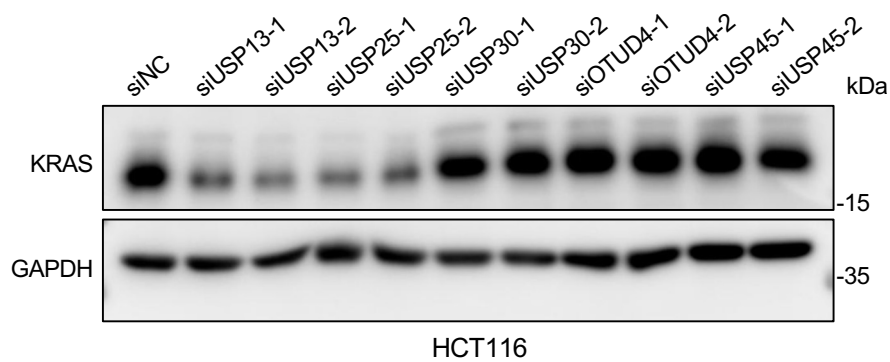**E**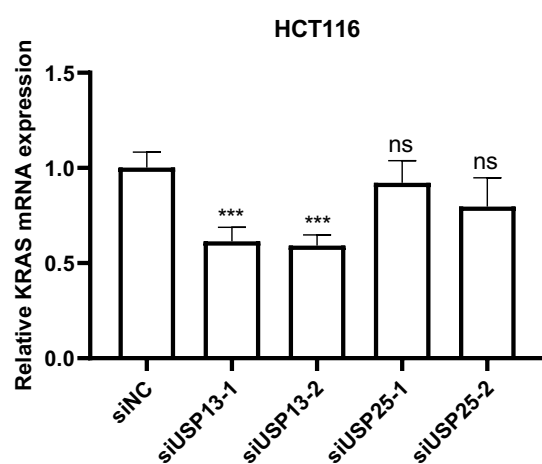**F**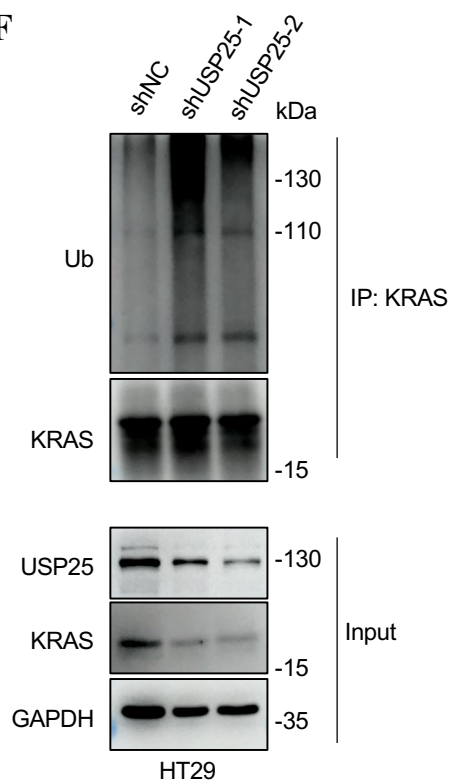**Figure S1**

Supplement: Supplementary file 3 — Figure S1 [file mmc3.pdf]

**A**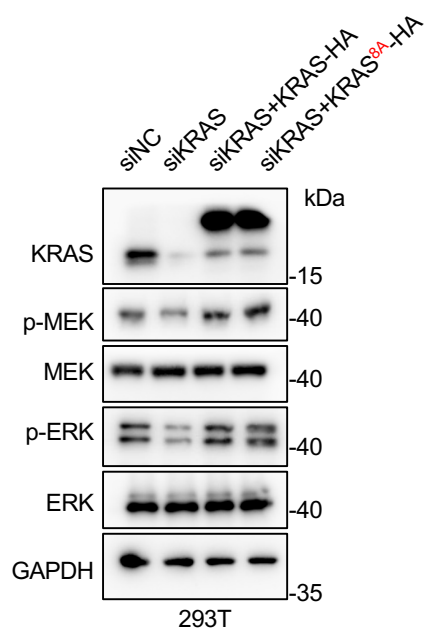**B**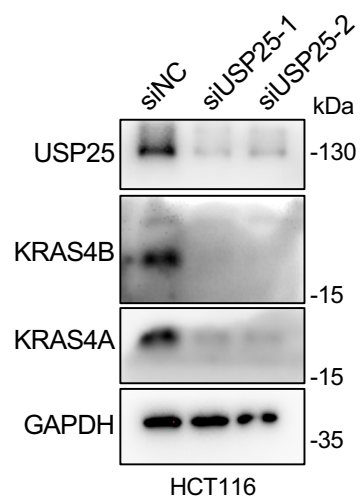**C**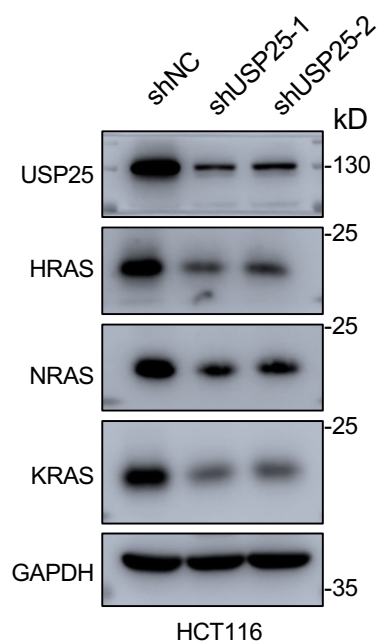**Figure S2**

Supplement: Figure S2 [file mmc4.pdf]

**A**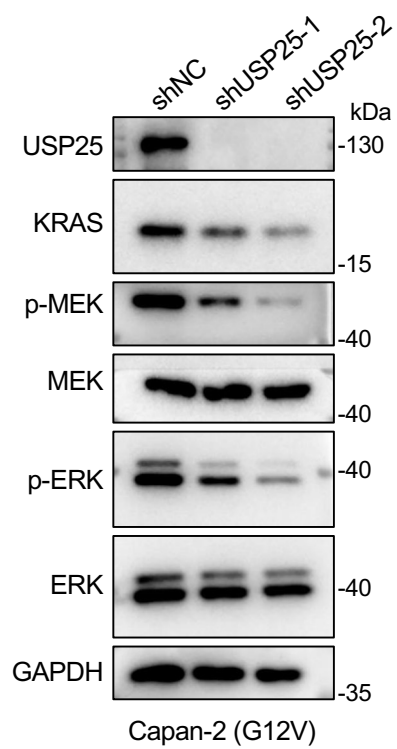**B**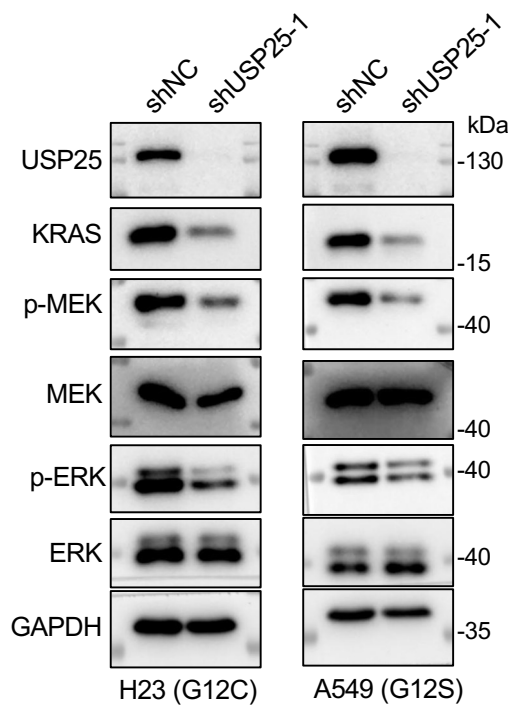**C**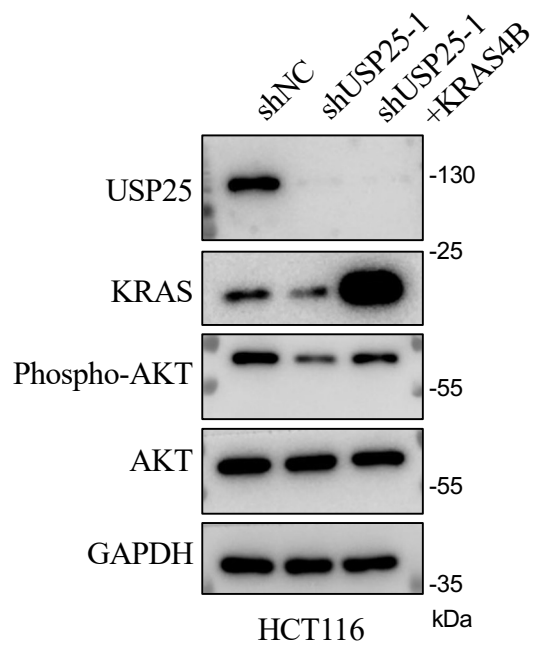**Figure S4**

Supplement: Figure S4 [file mmc6.pdf]

**A**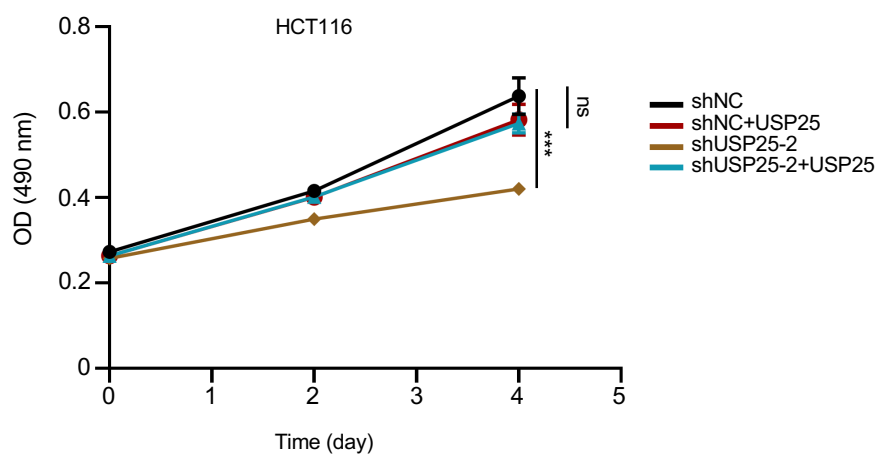**B**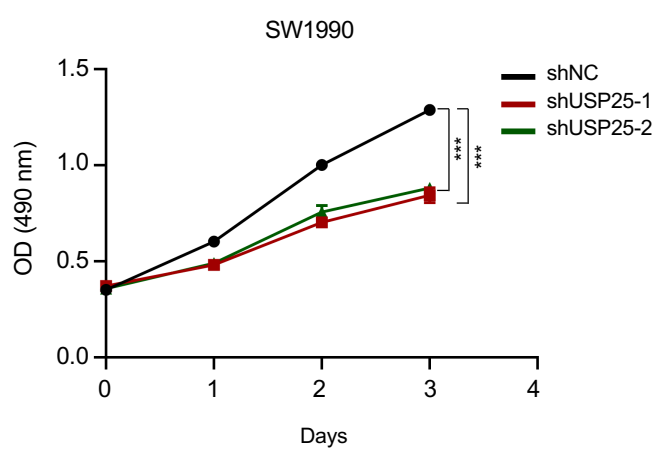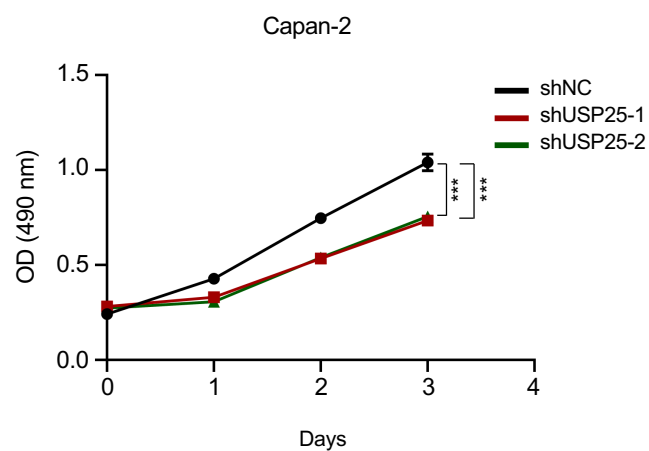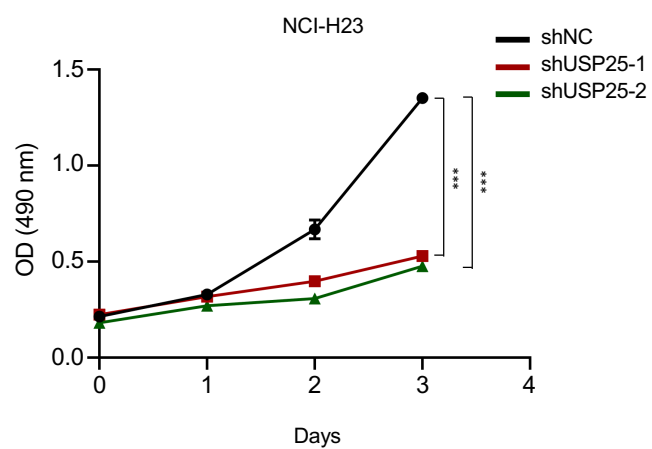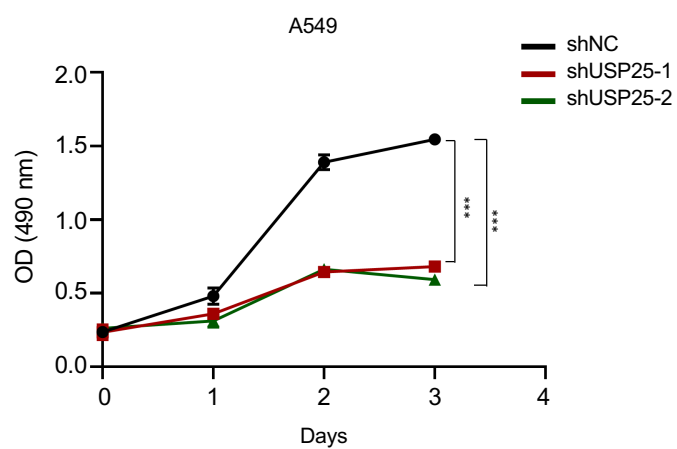**Figure S5**

C

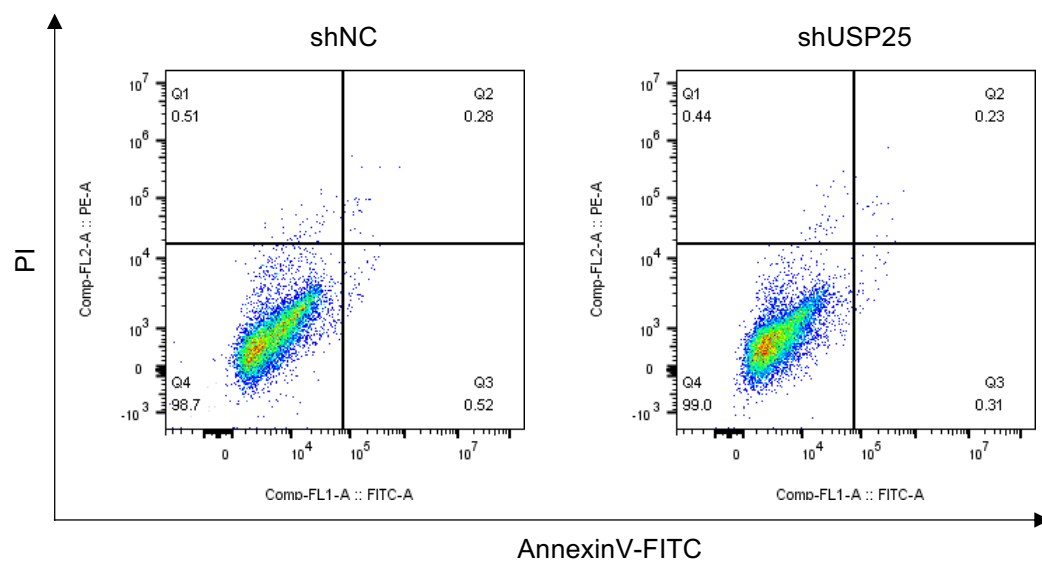

D

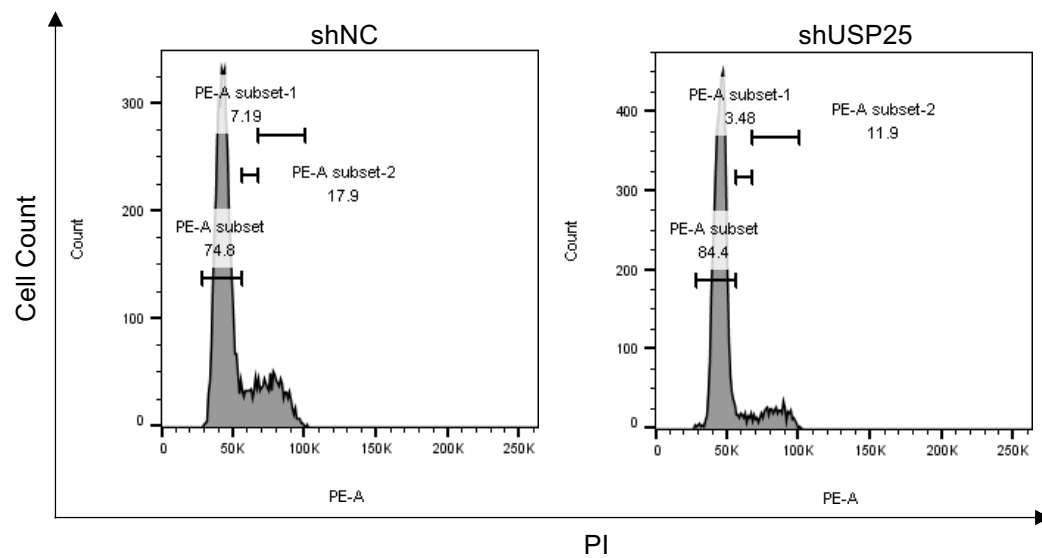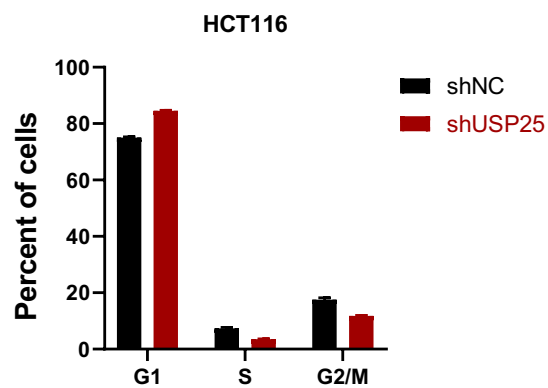

Figure S5

Supplement: Figure S5 [file mmc7.pdf]

**A**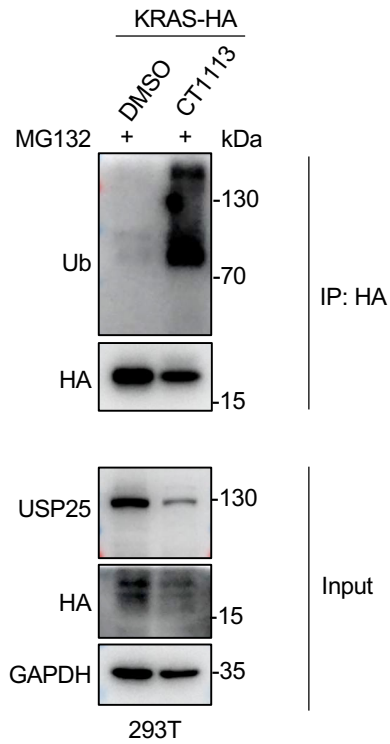**B**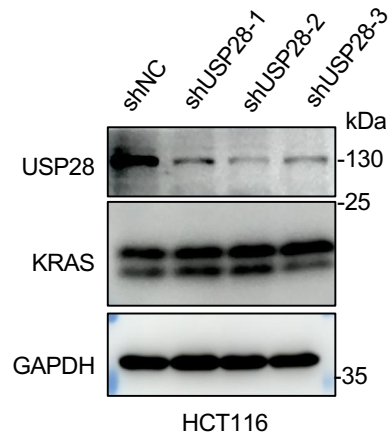**C**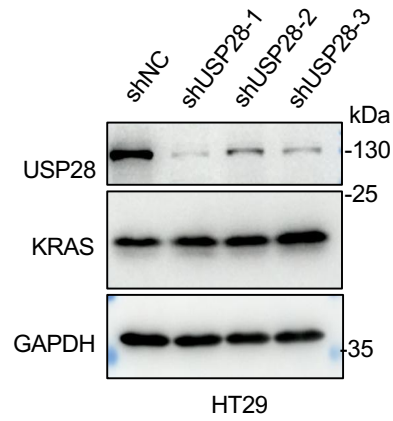**D**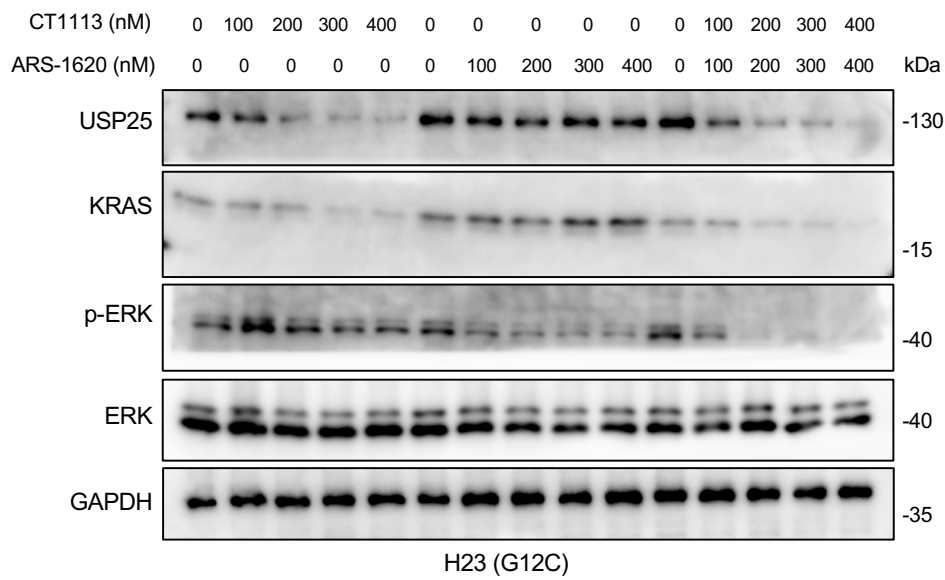**Figure S6**

E

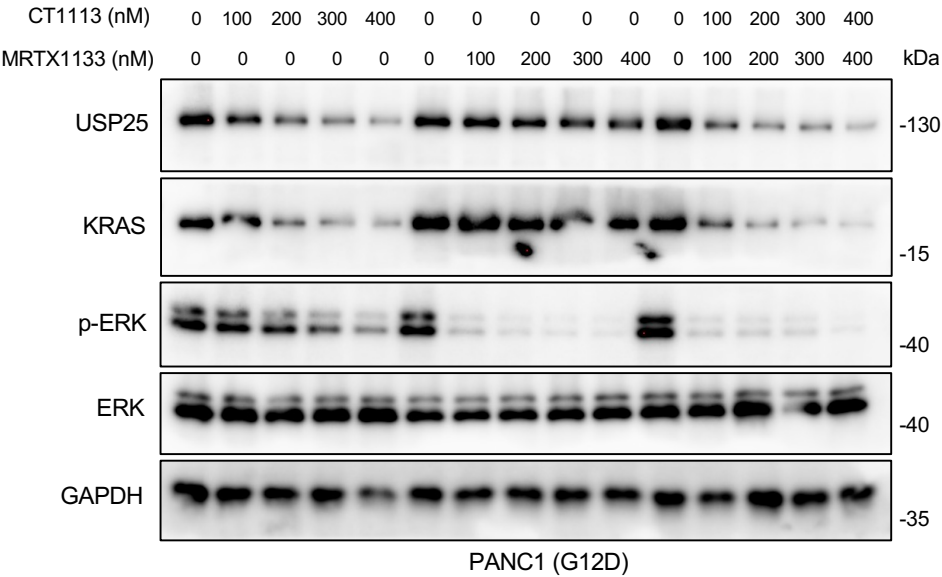

Figure S6

Supplement: Figure S6 [file mmc8.pdf]

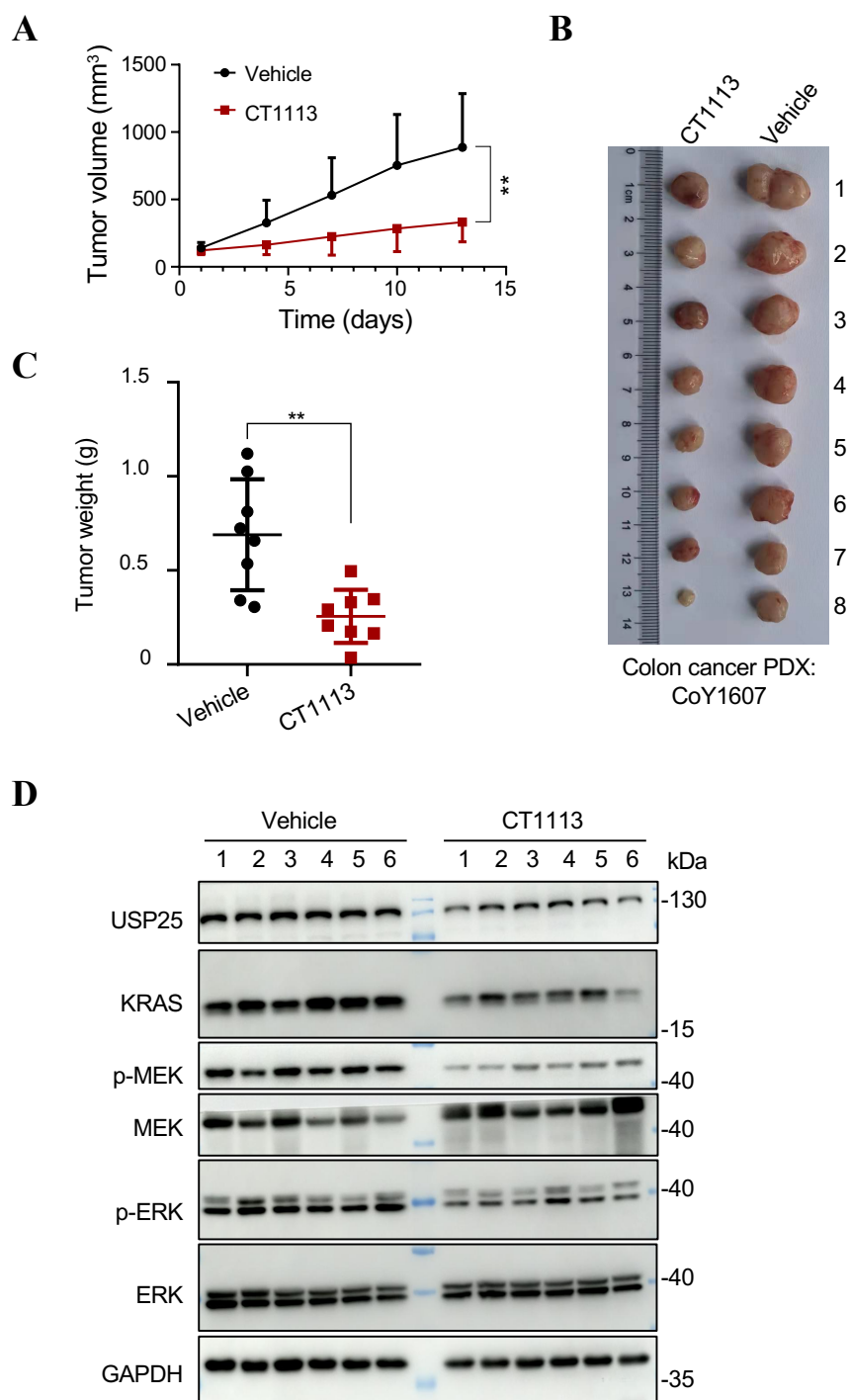

**Figure S7**

Supplement: Figure S7 [file mmc9.pdf]

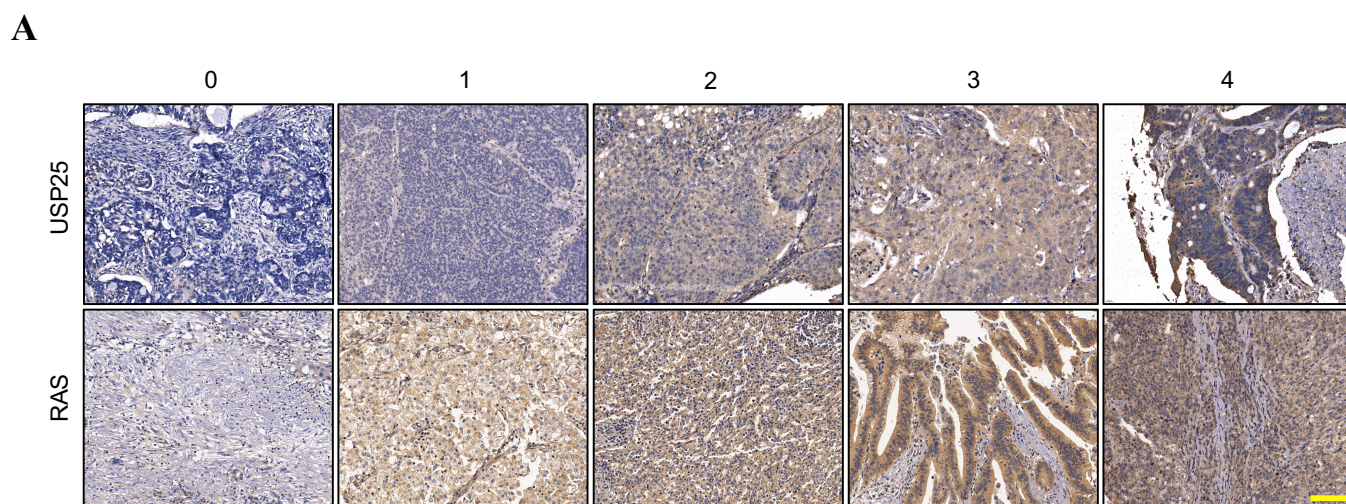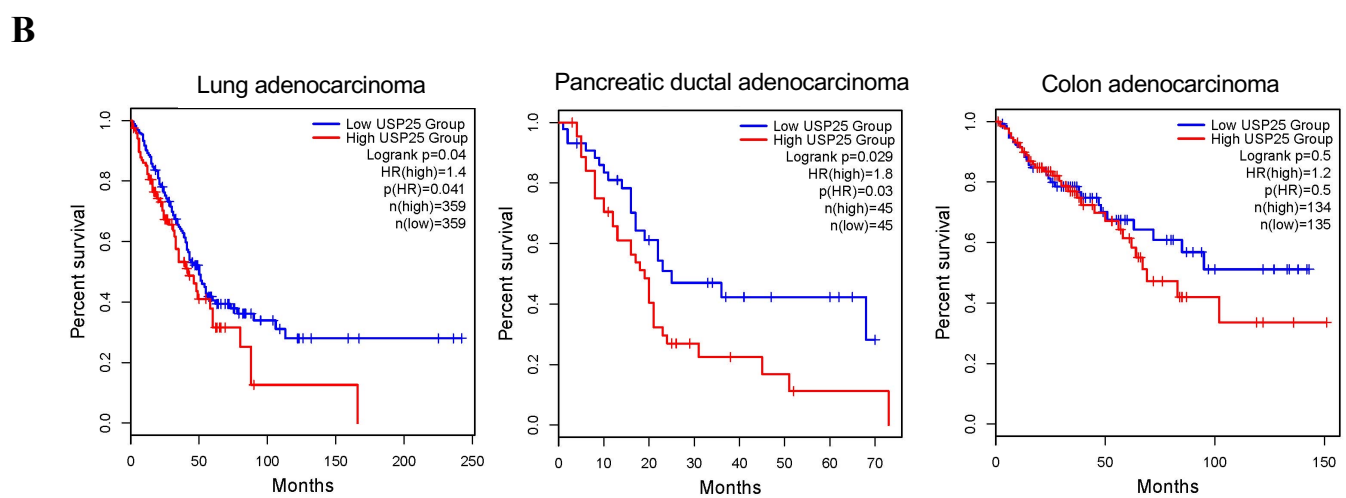

**Figure S8**

Supplement: Figure S8 [file mmc10.pdf]
