# Supplementary material for: USP25 maintains KRAS expression and inhibiting the deubiquitinase suppresses KRAS signaling in human cancer
Source: J Biol Chem. 2025 Jun 3;301(7):110337. doi: 10.1016/j.jbc.2025.110337 (PMC12269508; doi:10.1016/j.jbc.2025.110337)
Supplement: Figure S3 [file mmc5.pdf]

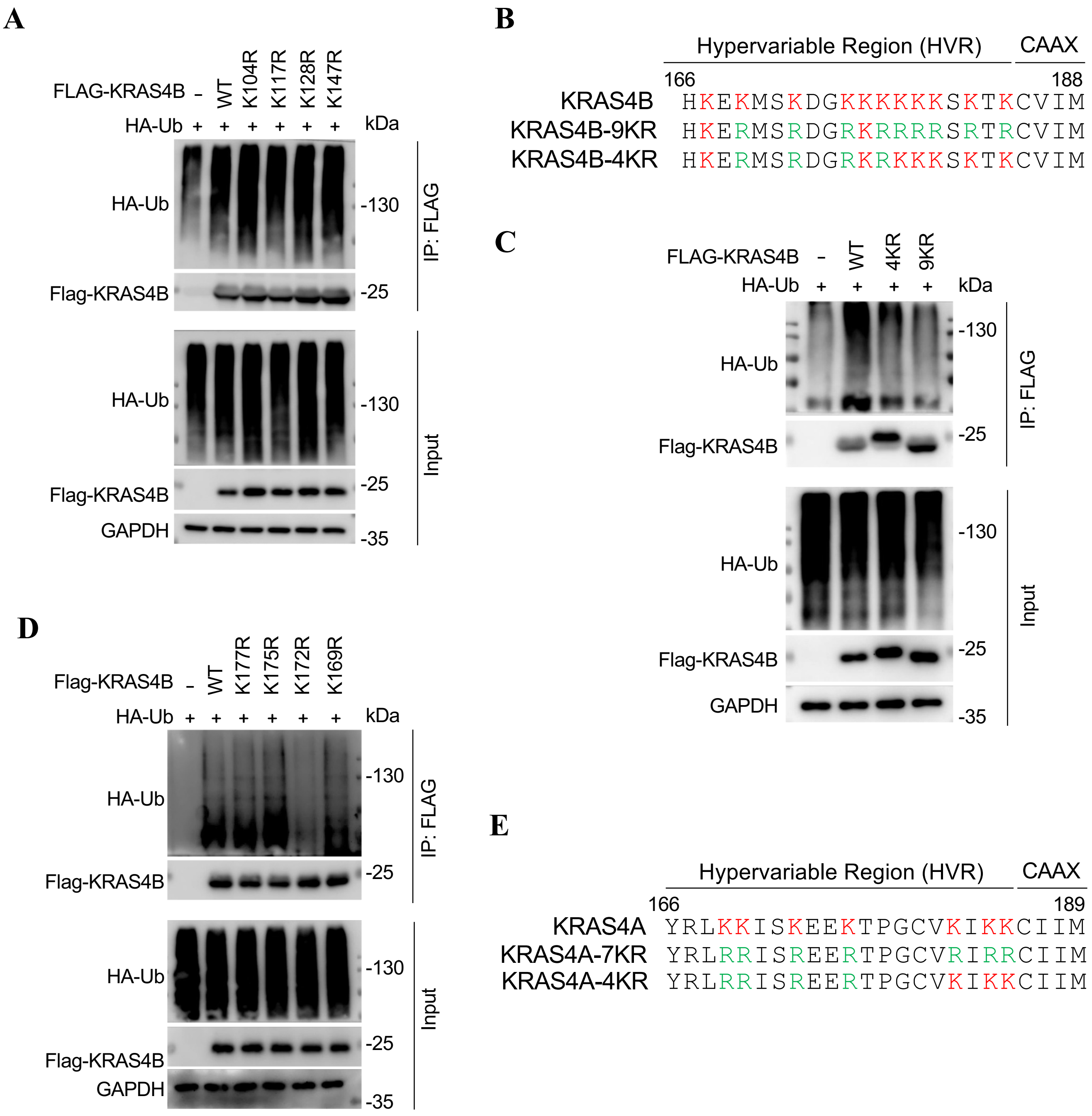
A
FLAG-KRAS4B – WT K104R K117R K128R K147R
HA-Ub + + + + + +
kDa
HA-Ub
-130
Flag-KRAS4B
-25
IP: FLAG
HA-Ub
-130
Flag-KRAS4B
-25
GAPDH
-35
Input
B
Hypervariable Region (HVR)
CAAX
166
188
KRAS4B HKEKMSKDGKKKKKKSKTKCVIM
KRAS4B-9KR HKERMSRDGRKRRRRSRTRCVIM
KRAS4B-4KR HKERMSRDGRKRKKKSKTKCVIM
C
FLAG-KRAS4B – WT 4KR 9KR
HA-Ub + + + +
kDa
HA-Ub
-130
Flag-KRAS4B
-25
IP: FLAG
HA-Ub
-130
Flag-KRAS4B
-25
GAPDH
-35
Input
D
Flag-KRAS4B – WT K177R K175R K172R K169R
HA-Ub + + + + + +
kDa
HA-Ub
-130
Flag-KRAS4B
-25
IP: FLAG
HA-Ub
-130
Flag-KRAS4B
-25
GAPDH
-35
Input
E
Hypervariable Region (HVR)
CAAX
166
189
KRAS4A YRLKKISKEEKTPGCVKIKKCIIM
KRAS4A-7KR YRLRRISREERTPGCVRIRRCIIM
KRAS4A-4KR YRLRRISREERTPGCVKIKKCIIM

Figure S3

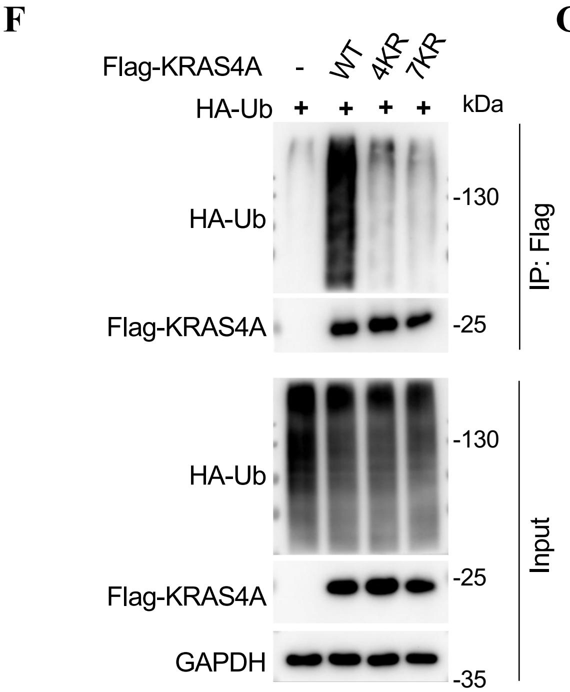
F
Flag-KRAS4A
-
WT
4KR
7KR
HA-Ub
+
+
+
+
kDa
HA-Ub
-130
Flag-KRAS4A
-25
IP: Flag
HA-Ub
-130
Flag-KRAS4A
-25
GAPDH
-35
Input

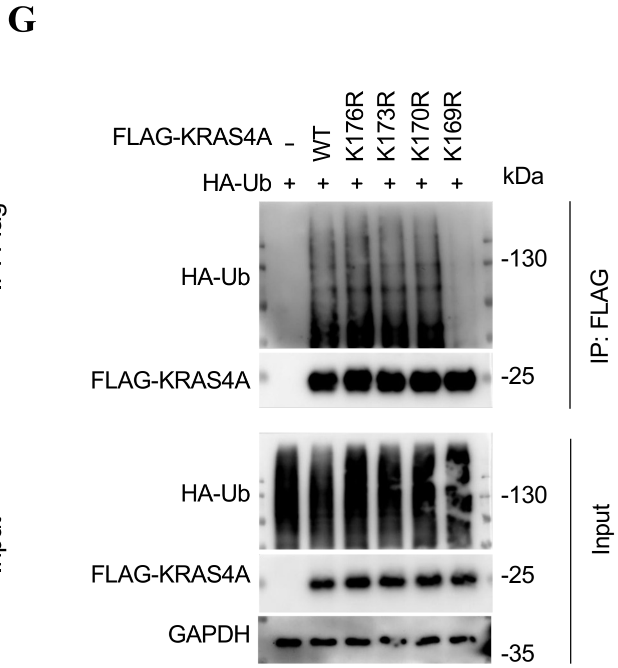
G
FLAG-KRAS4A
–
WT
K176R
K173R
K170R
K169R
HA-Ub
+
+
+
+
+
+
kDa
HA-Ub
-130
FLAG-KRAS4A
-25
IP: FLAG
HA-Ub
-130
FLAG-KRAS4A
-25
GAPDH
-35
Input

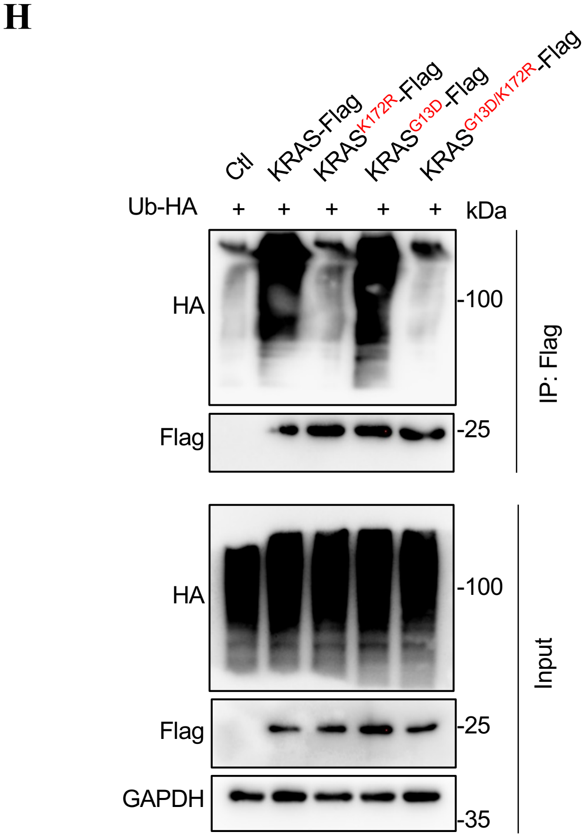
H
Ctl
KRAS-Flag
KRAS K172R -Flag
KRAS G13D -Flag
KRAS G13D/K172R -Flag
Ub-HA
+
+
+
+
+
kDa
HA
-100
Flag
-25
IP: Flag
HA
-100
Flag
-25
GAPDH
-35
Input

Figure S3
